# *Acmella oleracea* extracts as green pesticides against eight arthropods attacking stored products

**DOI:** 10.1007/s11356-023-28577-8

**Published:** 2023-08-05

**Authors:** Nickolas G. Kavallieratos, Eleonora Spinozzi, Constantin S. Filintas, Erifili P. Nika, Anna Skourti, Anna Maria E. Panariti, Marta Ferrati, Riccardo Petrelli, Massimo Ricciutelli, Simone Angeloni, Ettore Drenaggi, Alessia Sensini, Filippo Maggi, Angelo Canale, Giovanni Benelli

**Affiliations:** 1 Laboratory of Agricultural Zoology and Entomology, Department of Crop Science, Agricultural University of Athens, 75 Iera Odos str, Attica 11855 Athens, Greece; 2 Chemistry Interdisciplinary Project (ChIP) Research Center, School of Pharmacy, University of Camerino, Via Madonna Delle Carceri 9/B, 62032 Camerino, Italy; 3 Faculty of Biology, Institute of Zoology, University of Belgrade, Studentski trg 16, 11000 Belgrade, Serbia; 4 Department of Agriculture, Food and Environment, University of Pisa, Via del Borghetto 80, 56124 Pisa, Italy

**Keywords:** Adult, Grain protectant, Larva, Nymph, Plant-based pesticide, Stored-product pests

## Abstract

Developing sustainable control tools for managing noxious pests attacking stored foodstuffs is a timely research challenge. *Acmella oleracea* (L.) R. K. Jansen is a crop widely cultivated for its multiple usages on an industrial level. In this study, the extracts prepared with *A. oleracea* aerial parts were applied on wheat kernels for the management of eight important arthropod pests attacking stored products, i.e., *Cryptolestes ferrugineus, Tenebrio molitor, Oryzaephilus surinamensis, Trogoderma granarium, Tribolium castaneum, Tribolium confusum, Alphitobius diaperinus* (adults/larvae), and *Acarus siro* (adults/nymphs). Extraction of *A. oleracea* was optimized on the base of the yield and content of spilanthol and other *N*-alkylamides which were analysed by HPLC–DAD-MS. Two concentrations of *n*-hexane or methanol extracts (500 ppm and 1000 ppm), obtained through Soxhlet extraction, were tested to acquire mortality data on the above-mentioned pests after 4, 8, and 16 h and 1 to 7 days of exposure. Both extracts achieved complete mortality (100.0%) of *C. ferrugineus* adults. In the case of *A. diaperinus* adults, mortalities were very low at any concentrations of both extracts. In general, the *n*-hexane extract was more efficient than methanol extract against almost all species and stages. Considering both extracts, the susceptibility order, from most to least susceptible species/stage, was *C. ferrugineus* adults > *A. diaperinus* larvae > *C. ferrugineus* larvae > *T. granarium* adults > *T. molitor* larvae > *O. surinamensis* adults > *O. surinamensis* larvae > *T. confusum* larvae > *T. castaneum* larvae > *A. siro* adults > *T. molitor* adults > *A. siro* nymphs > *T. granarium* larvae > *T. castaneum* adults > *T. confusum* adults > *A. diaperinus* adults. Our research provides useful knowledge on the efficacy of *N*-alkylamides-rich *A. oleracea* extracts as grain protectants, pointing out the importance of targeting the most susceptible species/ developmental stages.

**Supplementary Information:**

The online version contains supplementary material available at 10.1007/s11356-023-28577-8.

## Introduction

**Fig. 1:**
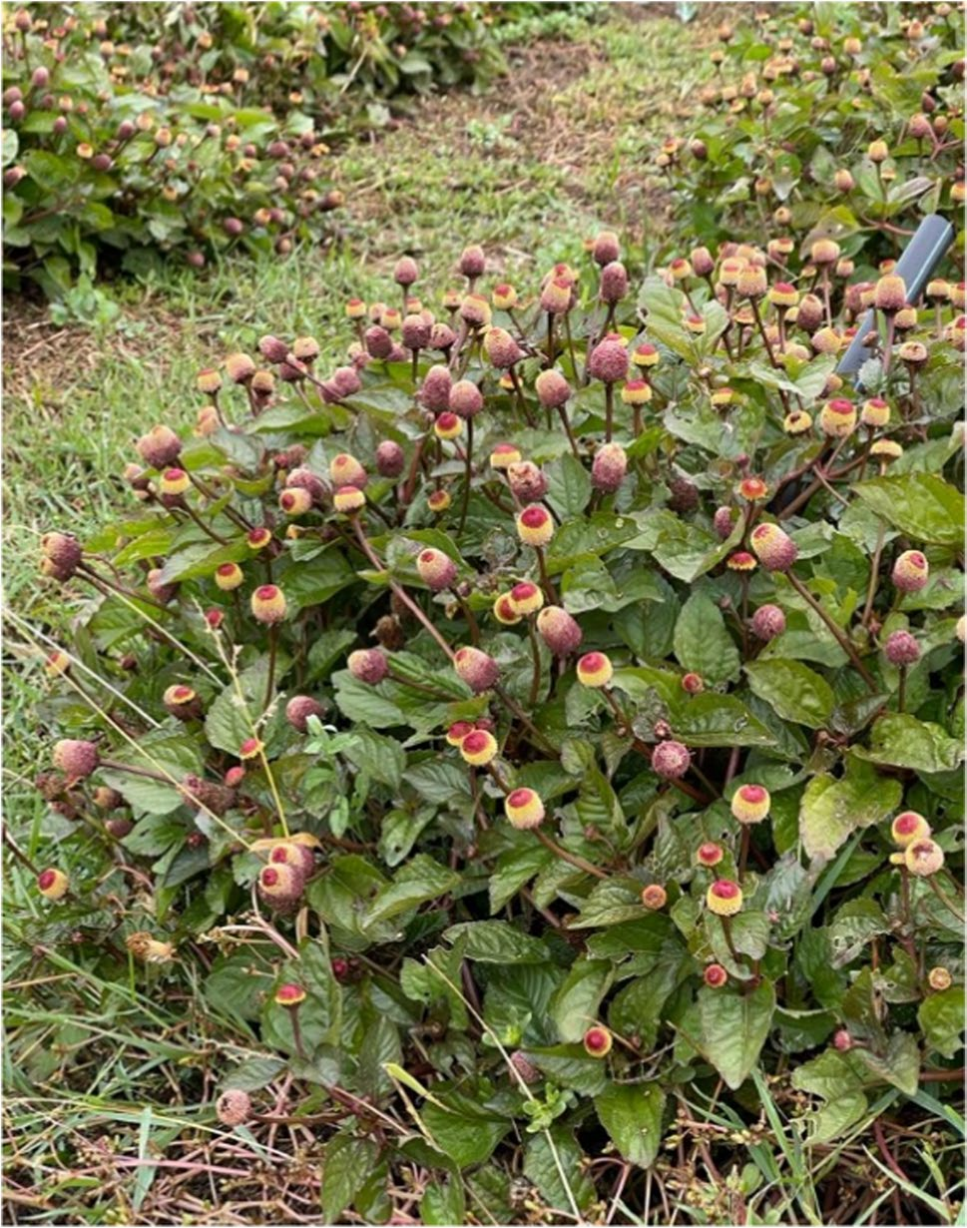
*Acmella oleracea* var. *oleracea* plant

The toothache plant, *Acmella oleracea* (L.) R. K. Jansen (Asterales: Asteraceae) (Fig. 1), is a cosmopolitan herb species, probably originating from Brazil, with distinct discoid, yellow, and red-tipped inflorescences (Jansen 1985; Uthpala and Navaratne 2021). The leaves and the flower buds of this plant are used for culinary purposes in Brazil, while it is industrially cultivated for its antimicrobial, cosmetic, insecticidal, and medicinal properties worldwide (Chung et al. 2008; Benelli et al. 2019; Rondanelli et al. 2020). Specifically, the consumption of *A. oleracea* stimulates saliva secretion and numbs the oral pain, hence its common name (Dubey et al. 2013). The extracts of this plant species exhibit potent analgesic, local anaesthetic, anti-inflammatory, and antioxidant properties, primarily used in modern dentistry (Matyushin and Evdokimova 2017). Its main bioactive compound is spilanthol, an isobutylamide (*N*-isobutylamide [(2*E*,6*Z*,8*E*)-*N*-isobutyl-2,6,8-decatrienamide]), which has been studied extensively for insecticidal purposes (Kadir et al. 1989; Sharma et al. 2012; Dubey et al. 2013; de Araújo et al. 2018; Araújo et al. 2020). Other compounds extracted from the entire plant are secondary metabolites, that include different flavonoids and phenolics with strong antioxidant properties (Sharma et al. 2022). The usual preparation of the extracts of *A. oleracea*, to be used as insecticides, is achieved employing *n*-hexane, methanol, and ethanol (Araújo et al. 2020). Studies related to the efficacy of *n*-hexane and ethanol extracts have documented high rates of toxicity against several agricultural insect pests (Moreno et al. 2012; Gouvêa et al. 2019; Spinozzi et al. 2022). For example, the *n*-hexane extract of *A. oleracea* killed all *Tuta absoluta* (Meyrick) (Lepidoptera: Gelechiidae) larvae after 6 h of exposure during contact toxicity trials, while the ethanol extract killed 88.3% at the same exposure (Moreno et al. 2012). Gouvêa et al. (2019) tested the efficacy of the ethanolic extract on *Lipaphis erysimi* (Kaltenbach) (Hemiptera: Aphididae) and *Myzus persicae* (Sulzer) (Hemiptera: Aphididae). The authors reported high mortality rates (90%) within 70 h, reduced fecundity, and no insecticidal activity against predators of both aphid species. Concerning the acaricidal activity, recent studies have evaluated the efficacy of different *A. oleracea* extracts on ectoparasitic acari (Cruz et al. 2016; Marchesini et al. 2020; de Oliveira et al. 2021). Cruz et al. (2016) presented high mortality rates, >90%, of the methanolic extract against larvae of *Rhipicephalus microplus* (Canestrini) (Acari: Ixodidae) and *Dermacentor nitens* (Neumann) (Acari: Ixodidae), caused by 1.6 and 6.2 mg/mL, respectively. Later, Marchesini et al. (2020) confirmed high mortality rates, caused by different concentrations of spilanthol in methanolic extracts of *A. oleracea,* against *R. microplus* adult females and larvae. In addition, the hydroethanolic extract of *A. oleracea* exhibits high mortality and low rates of ecotoxicity, making it a suitable alternative to commonly used synthetic compounds (de Araújo et al. 2018).

*Tribolium castaneum* (Herbst) (Coleoptera: Tenebrionidae), is a worldwide, small sized secondary pest (Hagstrum and Subramanyam 2009). It usually infests several commodities like cereals, nuts, coffee, spices, chocolate, with a preference to flours and processed foods (Kumar 2017). This species exhibits a long lifespan that causes economic damages due to its high population growth (Hill 2003; Skourti et al. 2019, 2022). The closely related species *Tribolium confusum* Jacquelin du Val (Coleoptera: Tenebrionidae) shares similar distributions and commodity preferences (Hill 2003; Rees 2004; Hagstrum and Subramanyam 2009; Hagstrum et al. 2013). *Tenebrio molitor* (L.) (Coleoptera: Tenebrionidae) is among the largest secondary stored-product coleopterans (12 – 18 mm), usually found in mills and grain storage facilities, mostly in temperate climates (Hill 2003; Rees 2004; Hagstrum and Subramanyam 2009; Nika et al. 2022). *Alphitobius diaperinus* (Panzer) (Coleoptera: Tenebrionidae) is a cosmopolitan secondary stored-product pest (Robinson 2005). It infests many commodities such as nuts, grains, tobacco, vegetables, and animal derivatives (Hagstrum and Subramanyam 2009). In poultry and hen houses it causes severe infestations as it can feed on decayed organic matter, like dead birds and mice (Hill 2003; Kumar 2017). *Oryzaephilus surinamensis* (L.) (Coleoptera: Silvanidae) is a global pest of storages, feeding on cereals, flours, and oilseeds (Rees 2004; Kumar 2017). It is a secondary pest with rapid growth rate in ideal thermal conditions (31.3 °C) and, in conjunction with its small size, can damage commodities undistinguished (Hill 2003; Nika et al. 2021). *Cryptolestes ferrugineus* (Stephens) (Coleoptera: Laemophloeidae) is an international secondary pest, commonly occurring in warm climates (Hagstrum et al. 2013). Usually, it occurs in grain storage facilities, infesting grains and their derivatives, nuts, oilseeds, and dried root crops (Hill 2003; Rees 2004). *Trogoderma granarium* (Everts) (Coleoptera: Dermestidae) is among the most destructive insects of stored commodities worldwide (Hill 2003; Rees 2004; Benelli et al. 2017; Kavallieratos et al. 2017). It has been classified as a quarantine pest in countries like the United States, Canada, Mexico, New Zealand, Morocco, and Belarus (EPPO 2022). *Trogoderma granarium* primarily infests grains, flours, oilseeds and has been reported in spices, herbs, dried fruit, and vegetables (Athanassiou et al. 2016; Kavallieratos et al. 2017, 2019a). Larvae can survive on harsh conditions, even enter a long-term diapausing state, making them hard to eradicate (Myers and Hagstrum 2012). *Acarus siro* (L.) (Sarcoptiformes: Acaridae) is a secondary pest, causing damages mostly to grain, flours, nuts, oilseeds, cheese, animal feed, vegetables, fruits, and herbs (Hagstrum et al. 2013).

Even though, the extracts of *A. oleracea* have been widely studied for their insecticidal properties against various insects of economic importance, with special reference to mosquitoes, houseflies, and moths (Kadir et al. 1989; Moreno et al. 2012; Sharma et al. 2012; Dubey et al. 2013; de Araújo et al. 2018; Araújo et al. 2020; Benelli et al. 2019), there are no data for stored-product pests. In an earlier research, Ogban et al. (2015) used plant powder from *A. oleracea* on maize against *Sitophilus zeamais* (Motschulsky) (Coleoptera: Curculionidae). Thus, the objective of the present study was to determine the pesticidal activity of *A. oleracea* *n*-hexane and methanol extracts, which derived from a screening of extraction solvents and extraction techniques, targeting the eight above-mentioned arthropods, as wheat protectants.

## Materials and methods

### Plant material

Flowering aerial parts of *A. oleracea* var. *oleracea* (Fig 1.) were purchased from the plantation of Dr. Ettore Drenaggi. The seeds were implanted between May and June 2021 in the Middle-end section of the Musone river’s riverbed, Castelfidardo (Italy) (43°27’16”N; 13°31’52”E). Aerial parts were dried at 38 °C for 72 h and then shredded with a plant grinder (Albrigi, mod. E0585, Stallavena, Verona Italy) with a 1.5 mm pore sieve. This material was used for the screening of extraction solvents and extraction techniques.

### Plant extraction

#### Screening of extraction solvents

For the screening of extraction solvents, dried and crushed aerial parts (20 g) were extracted in an ultrasound bath (Analogic ultrasonic bath Mod. AU-220, ARGOLAB, Carpi, Italy) at room temperature for 1 h using *n*-hexane, methanol, ethanol, dichloromethane, petroleum ether, and ethyl acetate (Sigma-Aldrich, Milan, Italy) always in a plant/solvent ratio of 1:10 (w/v). Subsequently, the various extracts obtained were filtered on a cotton filter and evaporated to dryness with a Rotavapor (Buchi Rotavapor R-200, Büchi Labortechnik AG, Flawil, Switzerland) at 40 °C. For each solvent, the extractions have been carried out in duplicate. The yields obtained, expressed in % w/w of dry extract (DE), are reported in Supplementary Material (Table S1).

#### Optimization of the extraction procedure

For the screening of various extraction techniques available in the authors’ laboratory, 20 g of plant material were extracted with the solvent that led to the highest extraction yield (Table S1) and spilanthol recovery from the plant (Table 1), always respecting the plant/solvent ratio of 1:10 (w/v). The extraction procedures are described below.

**Table 1:** Results of the first screening with different solvents used to prepare *A. oleracea* extracts

Solvent	Concentration (g/100 g DE^1^)^2^ ± SD^3^	RSD%^4^	Absolute amount of spilanthol extracted (g/100 g DB)^5^ ± SD
*n*-Hexane	20.9 ± 2.4a	11.7	0.6 ± 0.0b
Ethanol	11.4 ± 0.6c	5.5	0.4 ± 0.1b
Methanol	15.9 ± 2.6bc	16.4	1.3 ± 0.2a
Dichloromethane	17.7 ± 2.6ab	14.5	0.9 ± 0.2ab
Petroleum ether	19.7 ± 0.3ab	1.3	0.4 ± 0.0b
Ethyl acetate	16.5 ± 0.4abc	2.4	0.7 ± 0.0b
ANOVA *F*; *df*; *P*^6^	10.8496; 5; 0.0058		13.0059; 5; 0.0036

^1^DE, dry extract

^2^Mean concentration (g/100 g DE) represents the mean concentration of spilanthol found in each DE, and it is the mean of two independent analyses

^3^SD, standard deviation

^4^RSD%, relative SD

^5^Amount of spilanthol (g/100 g) extracted from dry biomass

^6^ANOVA parameters. Within a column, means followed by different letters are significantly different (Tukey's HSD test at *P* = 0.05)

Procedure A – Ultrasound bath: plant material and the solvent were placed in a flask, which was inserted in the ultrasound bath reported in section “Screening of extraction solvents”. The extraction was performed at room temperature for 2 different extraction times (1 and 3 h, respectively).

Procedure B – Ultrasound extractor: plant material and solvent were added in an ultrasound extractor (US2020, Albrigi Luigi) and the extraction was carried out with the program H + M (high power and mixing) for 1 h at room temperature.

Procedure C – Magnetic stirring: plant material and the solvent were placed in a flask and the extraction was carried out with magnetic stirring at 500 rpm at room temperature, varying the extraction times (1 and 3 h, respectively).

Procedure D - Soxhlet extraction: the plant material was extracted with the chosen solvent through a Soxhlet apparatus of 1 L capacity for 8 h.

Each extraction procedure has been carried out in duplicate and the extracts were evaporated as described in section “Screening of extraction solvents”. Moreover, the yields obtained (% w/w of DE) are reported in Supplementary Material (Table S1).

### Development of HPLC–DAD-MS method

#### Chemical and reagents

Spilanthol used for the analysis was obtained through a silica gel flash chromatography of the *A. oleracea* *n*-hexane extract*,* following the procedure by Spinozzi et al. (2021). HPLC-grade acetonitrile used for the analysis was acquired from Sigma-Aldrich (Milan, Italy).

#### Preparation of samples and standard solutions

The stock solutions containing 1700 and 850 mg/L of spilanthol were prepared in HPLC-grade methanol and stored at – 20 °C in glass vials till the chemical analysis. Further standard solutions were prepared by diluting the stock solutions to 170, 17, 1.7 and 85, 8.5 mg/L, respectively. The DEs were prepared at 1000 mg/L in acetonitrile. They were vortexed, for about 1 min and then further put in an ultrasound bath (section “Screening of extraction solvents”) for 5 min. Finally, they were filtered using a 0.2 μm syringeless filter.

### HPLC-DAD-MS conditions

The HPLC instrument was an Agilent 1100 series (Agilent Technologies, Santa Clara, CA, USA), consisting of a photodiode array detector (DAD), an autosampler, a binary solvent pump, and an ion-trap mass spectrometer (with electrospray ion source) LC/MSD Trap SL Agilent Technologies, controlled by LCMSD (Agilent, v.6.2) and ChemStation (Agilent, v.01.03) software. The chromatographic separation was conducted on a Luna C18 column (4.6 mm x 150 mm, i.d., particle size 5 μm), purchased from Phenomenex (Chesire, UK), which operated at 35 °C. Analysis was performed with a mobile phase consisting of acetonitrile and water. A linear gradient starting with 20% acetonitrile was set to reach 80% at 20 min and then hold for 20 min. Subsequently the column was reconditioned in 5 min and hold at this gradient for 15 min. The flow rate was 1 mL/min and the volume of injection was of 2 μL. The detection systems were DAD and ITMS. DAD was utilized for the quantification of spilanthol in the extracts deriving from the screenings and other *N*-alkylamides in the extracts selected for the biological assays. Chromatograms were recorded at 220 nm. All the compounds were confirmed by HPLC-MS analysis. The mass spectrometry system included an electrospray ionization (ESI) source functioning in positive ionization mode. Nitrogen was used as drying gas at 325 °C, at a flow rate of 12 L/min, and was also chosen as nebulizer gas at 70 psi. The capillary voltage was 3500 V. The mass scan was set between 50-900 *m/z* with a target mass of 222 *m/z*. 

## Method validation

Each solution was analysed in duplicate (*n* = 2). The calculation of the relative standard deviation percentage (RSD %) was conducted per sample to evaluate the precision of the obtained data. The HPLC-DAD-MS method was validated in terms of limits of detection (LODs), linearity, limits of quantification (LOQs), and precision. The linearity was evaluated by injecting standard solutions at various concentrations of spilanthol (1.7, 8.5, 17, 85, 170, 850, 1700 mg/L). Spilanthol calibration curve was constructed by plotting the analyte peak areas against the analyte concentrations, and this curve was used for the quantification of spilanthol and other *N*-alkylamides. The repeatability of the method was evaluated injecting each standard solution 3 times in HPLC in the same day (intraday), while the 850 mg/L solution was injected 3 times in 3 consecutive days (inter-day). The repeatability was evaluated in terms of relative standard deviation (RSD %) (Table S2). A signal-to-noise ratio (S/N) 3:1 was accepted to evaluate the LOD, while a signal-to-noise ratio 10:1 was considered for the determination of LOQ.

### Insect and mite species

*Cryptolestes ferrugineus, T. molitor, O. surinamensis, T. granarium, T. castaneum, T. confusum, A. diaperinus,* and *A. siro* used in this study were collected from mass-rearing of the Laboratory of Agricultural Zoology and Entomology, Agricultural University of Athens. *Tribolium* spp. and *C. ferrugineus* were cultured on brewer’s yeast (5%) mixed with wheat flour; *T. molitor* on wheat bran with chopped potatoes for enhanced moisture; *A. diaperinus* on wheat bran plus 25% yeast with chopped apple for enhanced moisture; *O. surinamensis* on broken wheat, brewer’s yeast and oat flakes (ratio 5: 1: 5); and *T. granarium* whole wheat (Hulasare et al. 2003; Sagheer et al. 2016; Kavallieratos et al. 2019b, 2020, 2022a, b; Nika et al. 2022). The rearing conditions were 30 °C, 65% relative humidity (RH), and darkness. Lastly, *A. siro* was reared on brewer’s yeast, wheat germ, and oat flakes, (ratio 1: 10: 10) at 25 °C, 80% RH, and darkness (Kavallieratos et al. 2018). Adult participants were randomly selected individuals, younger than 1 (*T. granarium*), 7 (*A. diaperinus*), and 14 (*C. ferrugineus, T. molitor, O. surinamensis, T. castaneum, T. confusum*) days old (Hulasare et al. 2003; Sagheer et al. 2016; Kavallieratos et al. 2019b, 2020, 2022a, b; Nika et al. 2021; Wang et al. 2021). Larval study subjects were between the 3^rd^ and the 4^th^ larval instar (*C. ferrugineus, O. surinamensis, T. castaneum, T. confusum*), between 10 and 14 mm long (*T. molitor*), shorter than 3 or 7 mm long (*T. granarium* and *A. diaperinus* respectively). *Acarus siro* was also selected randomly from reared individuals aged from 1 up to 21 days old. Nymphs were excluded from the adults by their morphology, i.e., short body setae (Hughes 1976; Nesvorna and Hubert 2014; Kavallieratos et al. 2018).

### Grain

*Triticum durum* Desf. (var. Claudio) (Poales: Poaceae), was used for the trials. Wheat kernels were clean, without pesticides or infestations. Before the bioassays, moisture content was measured to 12.9% with a mini-GAC plus moisture meter (Dickey-John Europe S.A.S., France).

### Bioassays

Preliminary trials were conducted at all arthropod (insect or mite) pests to select the two extract concentrations for the experiments: the lower 0.5 g extract/kg wheat (= 500 ppm) and the highest 1 g extract/kg wheat (= 1000 ppm). For the preparation of the solutions, a mixture of 0.125 g extract + 0.625 mL Tween 80 was vortexed until it was dissolved for 500 ppm. For 1000 ppm, 0.25 g extract + 1.25 mL Tween 80 were mixed and vortexed as aforementioned. Then, distilled water was added (5.375 and 4.75 mL for 500 and 1000 ppm respectively). The 6 mL solutions were separately sprayed on quantities of 0.25 kg wheat, laid each on tray, with a unique A BD-134K airbrush (Fengda, UK). Carrier control consisted of 0.25 mL Tween 80 mixed with 4.75 mL distilled water. A volume of 6 mL distilled water was used as control. Both carrier control and control were sprayed with different airbrushes on different quantites of 0.25 g wheat on different trays. Subsequently to the spraying, the lots of wheat were inserted to different 3-L glass jars and submitted to a 10 min handshake to further assure the homogeneous distribution of the extracts/controls to the whole commodity. Three samples of 10 g and 1 g were obtained from the lots, for the insect and mite pests respectively, weighted separately on a unique paper, on an electronic compact Precisa XB3200D balance (Alpha Analytical Instruments, Greece). Afterwards, the three 10 g and 1 g samples were transferred into different glass containers of 12.5 cm height + 7.5 cm diameter and of 6.0 cm height + 2.7 cm diameter, for the insect and mite species respectively. The first type of glass containers had lids with a circular whole of 1.5 cm diameter, which is covered with cloth, while the second type had drilled lids, to assure the aeration of their inside space. To stop pests from escaping the containers, polytetrafluoroethylene (Sigma-Aldrich Chemie GmbH, Germany) was used to polish the top inside part of them. Ten individuals of each arthropod pest/developmental stage were conveyed into the containers. Then, the containers with treated wheat and arthropods were put into incubators set at 30 °C, 65% RH (insects) or 25 °C, 80% RH (mite). Mortality data were acquired after 4, 8, and 16 h and 1 to 7 days, under a stereomicroscope (Olympus SZX9, Bacacos S.A., Greece). Individuals were regarded dead if no movement was tracked. For this purpose, a brush was utilized to slightly poke the pest individuals. The exact same preparation was repeated two more times for both concentrations/extracts/controls with fresh grains, arthropod individuals, and glass containers. In total, 8,640 pest individuals were acquired for the trials (3 replicates × 3 sub-replicates × 10 arthropod individuals × 6 concentrations/extracts (500 ppm, 1000 ppm, carrier control, control) × 16 arthropod species/developmental stages).

### Data analysis

Controls provided mortality that did not exceed 5% for all species and stages, hence data were not submitted to correction, but to log (x + 1) transformation (to normalize variance) (Zar 2014; Scheff and Arthur 2018). For each species/stage, a separate repeated-measures model analysis was conducted (Sall et al. 2001). Mortality, exposure, and concentration/extract were the response variable, repeated factor, and main effects, respectively. Their interactions were included in the analysis. Means were discreted by Tukey-Kramer test at 5% significance levels (Sokal and Rohlf 1995). Software JMP 16.2 was utilized for the analysis (SAS Institute Inc. 2021). Data about the optimization of extraction solvents and extraction techniques were analysed through one-way ANOVA followed by Tukey's HSD test (*p* < 0.05) (JMP 17, SAS).

## Results

### HPLC-DAD-MS quantification method

The linear regression equation obtained for spilanthol calibration curve was *y* = 6.5441*x* + 19.094, with a coefficient of determination (*R*^2^) of 1 (Fig. S1). The values obtained for LOQ and LOD were 0.153 mg/L and 0.046 mg/L, respectively.

### Screening results of *Acmella oleracea* extraction

The aim of this screening was the evaluation of spilanthol extraction capacity of different solvents, namely *n*-hexane, ethanol, methanol, dichloromethane, petroleum ether, and ethyl acetate chosen based on their different polarity. Firstly, this screening highlighted significant differences in extraction yields (Table S1). In this regard, the best extraction solvent resulted to be methanol with 11.0 ± 0.1% yield. Table 1 shows the HPLC-DAD results derived from the analysis of the DEs. In terms of spilanthol concentration, the best extract resulted to be *n*-hexane extract (20.9 ± 2.4 g/100 g DE), while ethyl acetate, methanol and ethanol extracts presented the lower concentration of the compound (16.5 ± 0.4, 15.9 ± 2.6, 11.4 ± 0.6 g/100 g DE, respectively). On the other hand, the analysis of the absolute amount of spilanthol extracted from the plant matrix revealed that the best spilanthol extractive solvent (Table 1) was methanol, with the *N*-alkylamide absolute amount extracted from the plant of 1.3%. The results of this first screening, led to the use of methanol as extractive solvent for the screening of different extraction techniques since it presented the highest spilanthol extraction capacity from the plant matrix.

The goal of this second screening was the identification of the best extraction technique leading to the highest recovery of spilanthol from the plant material. Different extraction techniques were screened: ultrasound bath, ultrasound extractor, magnetic stirring, and Soxhlet. This study led to the obtaining of different extraction yields (Table S1). The highest yield was obtained using Soxhlet (26%), while the yield obtained from the ultrasound extractor was of 7.6%. Conversely, the extract with the highest concentration of spilanthol was the one resulting from the extraction with the ultrasound extractor (12.3 ± 0.3 g/100 g DE) (Table 2). The absolute amount of spilanthol extracted from the plant matrix had a completely different trend. In fact, the technique leading to the highest recovery of spilanthol from the plant was Soxhlet (absolute amount extracted of 1.9%), while the ultrasound extractor led to the lowest recovery of the *N*-alkylamide from the plant (0.9%). In conclusion, from this second screening performed, the best technique for the highest recovery of spilanthol from the plant matrix resulted to be Soxhlet extraction.

For the evaluation of *A. oleracea* extracts efficacy against arthropods, two extracts were selected, applying Soxhlet extraction in both cases. Firstly, the methanol extract was chosen since methanol resulted the most efficient solvent in terms of spilanthol recovery from the plant. In addition, also a *n*-hexane extract was produced, since *n-*hexane was the solvent leading to the extract with the highest concentration of this *N*-alkylamide. Moreover, *n*-hexane is the most used solvent in previous entomological investigations of *A. oleracea* (Castro et al. 2014; Marchesini et al. 2020).

**Table 2:** Results of the screening of extraction techniques tested on *A. oleracea*

Extraction technique	Concentration (g/100 g DE^1^)^2^ ± SD^3^	RSD%^4^	Absolute amount of spilanthol extracted (g/100 g DB)^5^ ± SD
Ultrasound bath 1 h	11.8 ± 0.3a	2.7	1.2 ± 0.0bc
Ultrasound bath 3 h	11.3 ± 0.8a	7.4	1.3 ± 0.0b
Ultrasound extractor	12.3 ± 0.3a	2.5	0.9 ± 0.0c
Magnetic stirring 1 h	10.9 ± 0.5a	4.9	1.3 ± 0.1b
Magnetic stirring 3 h	11.3 ± 0.7a	6.3	1.4 ± 0.0b
Soxhlet	7.6 ± 0.3b	3.4	1.9 ± 0.2a
ANOVA *F*; *df*; *P*^6^	19.2937; 5; 0.0012		27.5567; 5; 0.0005

^1^DE, dry extract

^2^Mean concentration (g/100 g DE) represents the mean concentration of spilanthol found in each DE and it is the mean of two independent analyses

^3^SD, standard deviation

^4^RSD%, relative SD

^5^Amount of spilanthol (g/100 g) extracted from dry biomass

^6^ANOVA parameters. Within column, means followed by different letters are significantly different (Tukey's HSD test *P* = 0.05)

### *N*-Alkylamides quantification in *n*-hexane and methanolic extracts

For the extracts selected for the efficacy evaluation against pests, a quali-quantitative determination of the main *N*-alkylamides was performed by using the HPLC-DAD-MS method described in section “Development of HPLC–DAD-MS method”. A total of 6 *N*-alkylamides (Table 3) were identified through the evaluation of the MS spectra. For (*2Z*)-*N*-isobutyl-2-nonene-6,8-diynamide (A1), we confirmed its presence from [M + H]^+^ and [M + NH_4_]^+^ ions, which were 204.7 and 222.7 *m/z*, respectively (Fig. S2). The ions 232.6 and 254.6 *m/z* ([M + H]^+^ and [M + Na]^+^, respectively) confirmed the presence of (*2E*)-*N*-isobutyl-2-undecene-8,10-diynamide (A2) (Fig. S3); while the ions 222.5 and 244.5 *m/z* ([M + H]^+^ and [M + Na]^+^, respectively) were indicative of the presence of (*2E*,*6Z*,*8E*)-*N*-isobutyl-2,6,8-decatrienamide or spilanthol (A3) (Fig. S4).

**Table 3:** Results of the *N*-alkylamides HPLC–MS-DAD quantification in *A. oleracea* *n*-hexane and methanol extracts

	*N*-Alkylamide	*n*-Hexane extract			Methanol extract		
		Concentration (g/100 g DE^1^)^2^ ± SD^3^	RSD%^4^	Absolute amount of *N-*alkylamides extracted (g/100 g DB)^5^	Mean concentration (g/100 g DE) ± SD	RSD%	Absolute amount of *N-*alkylamides extracted (g/100 g DB)^e^
A1	(*2Z*)-*N*-Isobutyl-2-nonene-6,8-diynamide	0.4 ± 0.1	16.7	< 0.1	0.1 ± 0.0	0.0	< 0.1
A2	(*2E*)-*N*-Isobutyl-2-undecene-8,10-diynamide	0.3 ± 0.0	0.0	< 0.1	0.1 ± 0.0	0.0	< 0.1
A3	(*2E*,*6Z*,*8E*)-*N*-Isobutyl-2,6,8-decatrienamide (spilanthol)	24.3 ± 1.3	4.0	1.3	7.6 ± 0.3	3.4	1.9
A4	(*2E*,*7Z*)-*N*-Isobutyl-2,7-decadienamide	0.2 ± 0.0	0.0	< 0.1	0.1 ± 0.0	0.0	< 0.1
A5	(*2E*)-*N*-(2-Methylbutyl)-2-undecene-8,10-diynamide						
A6	(*2E*,*6Z*,*8E*)-*N*-(2-Methylbutyl)-2,6,8-decatrienamide	1.7 ± 0.3	13.0	0.1	0.5 ± 0.0	0.0	< 0.1

^1^DE, dry extract

^2^Average concentration (g/100 g DE) represents the average concentration of *N*-alkylamides found in each DE and it is the mean of two independent analyses

^3^SD, standard deviation

^4^RSD%, relative SD

^5^Amount of *N*-alkylamides (g/100 g) extracted from dry biomass

The *N*-alkylamides (*2E*,*7Z*)-*N*-isobutyl-2,7-decadienamide (A4) and (*2E*)-*N*-(2-methylbutyl)-2-undecene-8,10-diynamide (A5) coeluted from the column. For A4, 224.6 and 246.6 *m/z* (corresponding to [M + H]^+^ and [M + Na]^+^ ions) were detected; in addition 246.6 *m/z* corresponded also to the [M + H]^+^ ion of A5, for which also 268.6 *m/z* ([M + Na]^+^ ion) was detected. Finally, 236.6 and 258.6 *m/z* ([M + H]^+^ and [M + Na]^+^ ions) (Fig. S5) were detected for the *N*-alkylamide (*2E*,*6Z*,*8E*)-*N*-(2-methylbutyl)-2,6,8-decatrienamide (A6). From the quantification of the *N*-alkylamides in the *A. oleracea* extracts, the *n*-hexane extract resulted to be more concentrated. Spilanthol was the main compound in both *n*-hexane and methanol extracts, resulting in 24.3 ± 1.3 and 7.6 ± 0.3 g/100 g DE, respectively, followed by A6 (1.7 ± 0.3 and 0.5 ± 0.0 g/100 g DE, respectively). A1, A2 and A3 were detected in minor amounts in both extracts, as reported in Table 3. On the contrary, methanol extraction was more effective than *n*-hexane in terms of *N*-alkylamides recovery from the plant material, especially spilanthol (1.9 and 1.3%, respectively), as also demonstrated by the solvents screening reported in section “Grain”. The total amount of the other *N*-alkylamides recovered was < than 0.1%.

### Efficacy of *Acmella oleracea* extracts against arthropod pests

#### Adults and larvae of *Cryptolestes ferrugineus*

Concerning *C. ferrugineus* adults, main effects were significant between exposure intervals while exposure, exposure × extract and exposure × extract × concentration were significant within exposure intervals (Table 4). *n*-Hexane extract was very effective against *C. ferrugineus* adults killing 91.1% after 1 day of exposure to 1000 ppm (Table 5). A day after, 500 ppm and 1000 ppm of *n*-hexane extract caused the death to 96.7 and 100.0% of the individuals, respectively, while methanol extract did not achieved more than 70.0% mortality (1000 ppm). The 3^rd^ day, 500 ppm of *n*-hexane extract caused complete mortality (100.0%). For the 500 ppm and 1000 ppm of methanol extract, 100.0% deaths were achieved the 6^th^ day of the experimentation.

**Table 4:** MANOVA parameters for the main effects and associated interactions leading to the observed mortality rates of adults and larvae of *C. ferrugineus, T. molitor, O. surinamensis, T. granarium, T. castaneum, T. confusum,* and *A. diaperinus,* and *A. siro* adults and nymphs between and within exposure intervals (error *df* = 32)

		Between exposures				Within exposures			
		Intercept	Extract	Concentration	Extract × concentration	Exposure	Exposure × extract	Exposure × concentration	Exposure × extract × concentration
	DF	1	1	1	1	9	9	9	9
*C. ferrugineus* adults	*F*	7822.8	66.6	8.6	0.4	191.1	19.4	1.5	2.3
	*P*	< 0.01	< 0.01	0.01	0.52	< 0.01	< 0.01	0.22	0.05
*C. ferrugineus* larvae	*F*	5742.5	175.5	19.1	5.4	11,971.0	26.0	9.0	8.4
	*P*	< 0.01	< 0.01	< 0.01	0.03	< 0.01	< 0.01	< 0.01	< 0.01
*A. siro* adults	*F*	1156.8	4.1	18.2	1.9	884.4	0.6	7.9	1.1
	*P*	< 0.01	0.05	< 0.01	0.17	< 0.01	0.76	< 0.01	0.37
*A. siro* nymphs	*F*	1283.1	1.8	24.2	0.26	1013.1	3.7	7.8	0.4
	*P*	< 0.01	0.19	< 0.01	0.61	< 0.01	0.01	< 0.01	0.94
*O. surinamensis* adults	*F*	4691.9	15.0	47.5	6.2	2364.6	5.5	9.2	6.1
	*P*	< 0.01	< 0.01	< 0.01	0.02	< 0.01	< 0.01	< 0.01	< 0.01
*O. surinamensis* larvae	*F*	4752.2	71.5	39.5	< 0.1	4458.2	10.0	6.7	1.1
	*P*	< 0.01	< 0.01	< 0.01	0.87	< 0.01	< 0.01	< 0.01	0.37
*T. granarium* adults	*F*	3597.6	11.3	29.6	7.3	5082.8	11.0	8.6	6.9
	*P*	< 0.01	< 0.01	< 0.01	0.01	< 0.01	< 0.01	< 0.01	< 0.01
*T. granarium* larvae	*F*	444.1	6.4	18.2	1.6	51.1	4.6	2.2	1.8
	*P*	< 0.01	0.02	< 0.01	0.21	< 0.01	< 0.01	0.06	0.13
*T. castaneum* adults	*F*	89.3	0.6	5.9	0.1	10.7	2.6	0.6	1.3
	*P*	< 0.01	0.47	0.02	0.74	< 0.01	0.03	0.82	0.30
*T. castaneum* larvae	*F*	1481.3	60.0	55.6	2.5	1278.2	5.7	6.5	3.2
	*P*	< 0.01	< 0.01	< 0.01	0.12	< 0.01	< 0.01	0.01	0.01
*T. confusum* adults	*F*	25.2	4.6	5.5	0.3	3.3	1.4	1.6	0.6
	*P*	< 0.01	0.05	0.03	0.58	0.01	0.26	0.17	0.81
*T. confusum* larvae	*F*	2020.9	0.5	4.2	0.7	1340.1	11.4	2.0	2.2
	*P*	< 0.01	0.51	0.05	0.42	< 0.01	< 0.01	0.09	0.06
*T. molitor* adults	*F*	472.8	108.6	4.2	1.3	51.7	12.2	0.7	0.5
	*P*	< 0.01	< 0.01	0.05	0.26	< 0.01	< 0.01	0.69	0.88
*T. molitor* larvae	*F*	5399.2	19.5	164.6	0.2	7192.1	14.1	26.6	18.7
	*P*	< 0.01	< 0.01	< 0.01	0.67	< 0.01	< 0.01	< 0.01	< 0.01
*A. diaperinus* adult	*F*	2.9	1.9	1.9	1.3	0.5	0.2	0.2	0.2
	*P*	0.10	0.17	0.17	0.27	0.86	0.99	0.99	0.99
*A. diaperinus* larvae	*F*	4555.2	1.4	21.7	3.9	11,241.0	2.7	7.9	5.4
	*P*	< 0.01	0.25	< 0.01	0.06	< 0.01	0.03	< 0.01	< 0.01

**Table 5:** Mean (%) mortality ± standard error (SE) of *C. ferrugineus* adults and larvae after 4 h, 8 h, 16 h, and 1–7 days on wheat treated with *A. oleracea* *n*-hexane and methanol extracts at two different concentrations

Adults	*n*-Hexane extract 500 ppm	*n*-Hexane extract 1000 ppm	Methanol extract 500 ppm	Methanol extract 1000 ppm	*F*	*P*
4 h	0.0 ± 0.0Db	3.3 ± 1.7Ca	0.0 ± 0.0Cb	0.0 ± 0.0Eb	4.0	0.02
8 h	12.2 ± 2.2Ca	23.3 ± 4.1Ba	2.2 ± 1.5Cb	3.3 ± 1.7Db	10.1	< 0.01
16 h	40.0 ± 3.7Ba	55.6 ± 6.5Aa	5.6 ± 2.9Cc	12.2 ± 1.5Cb	26.2	< 0.01
1 day	73.3 ± 4.7Aa	91.1 ± 3.5Aa	17.8 ± 4.3Bb	24.4 ± 2.4Bb	22.5	< 0.01
2 days	96.7 ± 1.7Aa	100.0 ± 0.0Aa	60.0 ± 7.3Ab	70.0 ± 5.3Ab	13.9	< 0.01
3 days	100.0 ± 0.0Aa	100.0 ± 0.0Aa	83.3 ± 6.0Ab	92.2 ± 4.3Aab	4.2	0.01
4 days	100.0 ± 0.0Aa	100.0 ± 0.0Aa	93.3 ± 3.7Ab	100.0 ± 0.0Aa	3.1	0.04
5 days	100.0 ± 0.0A	100.0 ± 0.0A	97.8 ± 1.5A	100.0 ± 0.0A	2.3	0.10
6 days	100.0 ± 0.0A	100.0 ± 0.0A	100.0 ± 0.0A	100.0 ± 0.0A	-	-
7 days	100.0 ± 0.0A	100.0 ± 0.0A	100.0 ± 0.0A	100.0 ± 0.0A	-	-
*F*	211.1	45.0	71.1	162.8		
*P*	< 0.01	< 0.01	< 0.01	< 0.01		
Larvae						
4 h	0.0 ± 0.0C	0.0 ± 0.0D	0.0 ± 0.0D	0.0 ± 0.0D	-	-
8 h	0.0 ± 0.0C	1.1 ± 1.1D	0.0 ± 0.0D	0.0 ± 0.0D	1.0	0.41
16 h	1.1 ± 1.1Cb	20.0 ± 3.3Ca	0.0 ± 0.0Db	0.0 ± 0.0Db	86.2	< 0.01
1 day	16.7 ± 4.1Bb	42.2 ± 4.7Ba	0.0 ± 0.0Dc	1.1 ± 1.1Dc	61.0	< 0.01
2 days	52.2 ± 5.2Aa	75.6 ± 6.5Aa	8.9 ± 3.1Cb	12.2 ± 2.8Cb	16.3	< 0.01
3 days	73.3 ± 3.7Aa	87.8 ± 5.7Aa	22.2 ± 3.2Bb	23.3 ± 2.9Bb	48.0	< 0.01
4 days	87.8 ± 2.8Aa	91.1 ± 6.1Aa	45.6 ± 2.9Abb	54.4 ± 3.8Ab	28.3	< 0.01
5 days	93.3 ± 2.4Aa	95.6 ± 3.4Aa	65.6 ± 4.4Ab	67.8 ± 2.8Ab	18.5	< 0.01
6 days	96.7 ± 2.4Aa	98.9 ± 1.1Aa	81.1 ± 4.8Ab	92.2 ± 2.8Aab	6.4	< 0.01
7 days	96.7 ± 2.4Aab	98.9 ± 1.1Aa	86.7 ± 4.7Ab	100.0 ± 0.0Aa	4.8	0.01
*F*	195.3	244.5	143.9	155.6		
*P*	< 0.01	< 0.01	< 0.01	< 0.01		

Within each row, means followed by the same lowercase letter are not significantly different (*df* = 3, 35; Tukey's HSD test at *P* = 0.05). Within each column, means followed by the same uppercase letter are not significantly different (*df* = 9, 89; Tukey's HSD test at *P* = 0.05). Where no letters exist, no significant differences were recorded. Where dashes exist, no statistical analysis was performed

For *C. ferrugineus* larvae, between and within exposure intervals all main effects and their interactions were significant (Table 4). The 2^nd^ day both concentrations of the *n*-hexane extract achieved moderate efficacy, reaching 52.2% (500 ppm) and 75.6% (1000 ppm) mortality (Table 5). At the 7^th^ day, 500 ppm and 1000 ppm of *n*-hexane extract killed 96.7 and 98.9% of the larvae, respectively. In addition, the 1000 ppm of methanol caused the death to all exposed larvae at the same exposure period. Methanol extract at 500 ppm caused 86.7% mortality at the end of the trials.

#### Adults and nymphs of *Acarus siro*

As far as *A. siro* adults are concerned, main effects were significant between exposure intervals, while only exposure and exposure × concentration were significant within exposure intervals (Table 4). For 1 day, no mortality was noticed at all tested extracts or concentrations (Table 6). Both extracts tested at 1000 ppm killed >50.0% after 5 days of exposure. At the end of the trials, 100.0% (1000 ppm of *n*-hexane extract) and 94.4% (1000 ppm of methanol extract) of the adults were dead, while 500 ppm of the extracts provided moderate mortalities i.e., 45.6% (*n*-hexane extract) and 57.8% (methanol extract).

**Table 6:** Mean (%) mortality ± standard error (SE) of *A. siro* adults and nymphs after 4 h, 8 h, 16 h, and 1–7 days on wheat treated with *A. oleracea* *n*-hexane and methanol extracts at two different concentrations

Adults	*n*-Hexane extract 500 ppm	*n*-Hexane extract 1000 ppm	Methanol extract 500 ppm	Methanol extract 1000 ppm	*F*	*P*
4 h	0.0 ± 0.0C	0.0 ± 0.0D	0.0 ± 0.0D	0.0 ± 0.0D	-	-
8 h	0.0 ± 0.0C	0.0 ± 0.0D	0.0 ± 0.0D	0.0 ± 0.0D	-	-
16 h	0.0 ± 0.0C	0.0 ± 0.0D	0.0 ± 0.0D	0.0 ± 0.0D	-	-
1 day	0.0 ± 0.0C	0.0 ± 0.0D	0.0 ± 0.0D	0.0 ± 0.0D	-	-
2 days	2.2 ± 1.5BC	3.3 ± 1.7D	3.3 ± 1.7CD	6.7 ± 1.7C	1.4	0.26
3 days	4.4 ± 2.4BC	13.3 ± 3.3C	8.9 ± 2.6C	12.2 ± 2.8BC	2.5	0.07
4 days	7.8 ± 2.7Bb	26.7 ± 2.9Ba	16.7 ± 2.9Ba	25.6 ± 6.5Ba	8.4	< 0.01
5 days	27.8 ± 3.6Ab	55.6 ± 5.0ABa	45.6 ± 5.3Ab	57.8 ± 7.4Aa	5.9	< 0.01
6 days	35.6 ± 3.8Ac	83.3 ± 2.9Aa	52.2 ± 5.2Ab	80.0 ± 4.4Aa	21.2	< 0.01
7 days	45.6 ± 5.0Ab	100.0 ± 0.0Aa	57.8 ± 6.2Ab	94.4 ± 2.9Aa	19.6	< 0.01
*F*	42.7	106.4	69.7	114.7		
*P*	< 0.01	< 0.01	< 0.01	< 0.01		
Nymphs						
4 h	0.0 ± 0.0C	0.0 ± 0.0C	0.0 ± 0.0D	0.0 ± 0.0D	-	-
8 h	0.0 ± 0.0C	0.0 ± 0.0C	0.0 ± 0.0D	0.0 ± 0.0D	-	-
16 h	0.0 ± 0.0C	0.0 ± 0.0C	0.0 ± 0.0D	0.0 ± 0.0D	-	-
1 day	0.0 ± 0.0C	0.0 ± 0.0C	0.0 ± 0.0D	0.0 ± 0.0D	-	-
2 days	0.0 ± 0.0C	0.0 ± 0.0C	0.0 ± 0.0D	0.0 ± 0.0D	-	-
3 days	4.4 ± 1.8Bb	13.3 ± 2.4Ba	5.6 ± 2.4Cb	13.3 ± 3.3Ca	2.9	0.05
4 days	7.8 ± 1.5B	20.0 ± 3.3B	16.7 ± 3.7B	30.0 ± 5.8BC	2.6	0.07
5 days	15.6 ± 1.8Ac	36.7 ± 4.1Aab	24.4 ± 4.1ABbc	48.9 ± 4.8ABa	14.4	< 0.01
6 days	24.4 ± 2.9Ab	47.8 ± 4.0Aa	26.7 ± 4.4ABb	57.8 ± 4.3Aa	13.4	< 0.01
7 days	26.7 ± 2.4Ac	50.0 ± 3.7Ab	38.9 ± 2.6Ab	68.9 ± 6.1Aa	24.0	< 0.01
*F*	60.4	209.1	63.7	94.9		
*P*	< 0.01	< 0.01	< 0.01	< 0.01		

Within each row, means followed by the same lowercase letter are not significantly different (*df* = 3, 35; Tukey's HSD test at *P* = 0.05). Within each column, means followed by the same uppercase letter are not significantly different (*df* = 9, 89; Tukey's HSD test at *P* = 0.05). Where no letters exist, no significant differences were recorded. Where dashes exist, no statistical analysis was performed

For *A. siro* nymphs, only concentration was significant between exposure intervals (Table 4). Within exposure intervals, all main effects were important. No mortality was observed for 2 days, for all tested extracts and concentrations (Table 6). Moderate efficacy was recorded at the termination of the bioassays, not exceeding 50.0% for 1000 ppm of *n*-hexane extract and 68.9% for 1000 ppm of methanol extract.

#### Adults and larvae of *Oryzaephilus surinamensis*

Between and within exposure intervals, all main effects and interactions were significant for *O. surinamensis* adults (Table 4). Both extracts tested at 1000 ppm provided ~50.0% mortality the 2^nd^ day of the trials, while the 5^th^ day ~90% (Table 7). At the termination of the bioassays, the *n*-hexane extract killed 90.0% of the adults and methanol extract 97.8%. The concentration of 500 ppm also provided high mortality levels reaching 87.8% and 82.2%, for *n*-hexane and methanol extracts, respectively.

**Table 7:** Mean (%) mortality ± standard error (SE) of *O. surinamensis* adults and larvae after 4 h, 8 h, 16 h, and 1–7 days on wheat treated with *A. oleracea* *n*-hexane and methanol extracts at two different concentrations

Adults	*n*-Hexane extract 500 ppm	*n*-Hexane extract 1000 ppm	Methanol extract 500 ppm	Methanol extract 1000 ppm	*F*	*P*
4 h	0.0 ± 0.0D	0.0 ± 0.0D	0.0 ± 0.0C	0.0 ± 0.0C	-	-
8 h	0.0 ± 0.0Db	3.3 ± 1.7Ca	0.0 ± 0.0Cb	0.0 ± 0.0Cb	4.0	0.02
16 h	0.0 ± 0.0Db	17.8 ± 2.8Ba	0.0 ± 0.0Cb	2.2 ± 1.5BCb	50.2	< 0.01
1 day	7.8 ± 2.8Cbc	30.0 ± 5.0Ba	0.0 ± 0.0Cc	8.9 ± 3.9Bb	13.7	< 0.01
2 days	21.1 ± 4.2Bb	58.9 ± 5.4Aa	24.4 ± 6.5Bb	47.8 ± 3.6Aa	7.6	< 0.01
3 days	42.2 ± 4.0ABb	75.6 ± 7.3Aa	63.3 ± 5.0Aa	67.8 ± 5.2Aa	7.9	< 0.01
4 days	61.1 ± 4.8Ab	86.7 ± 5.3Aa	77.8 ± 4.9Aa	87.8 ± 3.6Aa	7.3	< 0.01
5 days	83.3 ± 5.8A	90.0 ± 5.0A	78.9 ± 4.6A	92.2 ± 2.8A	1.7	0.19
6 days	87.8 ± 6.4A	90.0 ± 5.0A	80.0 ± 4.7A	94.4 ± 1.8A	1.5	0.23
7 days	87.8 ± 6.4A	90.0 ± 5.0A	82.2 ± 4.3A	97.8 ± 1.5A	1.7	0.18
*F*	137.1	109.0	266.2	100.5		
*P*	< 0.01	< 0.01	< 0.01	< 0.01		
Larvae						
4 h	0.0 ± 0.0E	0.0 ± 0.0D	0.0 ± 0.0D	0.0 ± 0.0E	-	-
8 h	0.0 ± 0.0E	1.1 ± 1.1D	0.0 ± 0.0D	1.1 ± 1.1E	0.7	0.58
16 h	3.3 ± 2.4Eb	13.3 ± 2.4Ca	0.0 ± 0.0Db	3.3 ± 1.7DEb	10.0	< 0.01
1 day	17.8 ± 3.6Dab	28.9 ± 3.5Ba	2.2 ± 1.5Dc	6.7 ± 1.7Dbc	14.0	< 0.01
2 days	27.8 ± 4.0CDab	53.3 ± 4.1ABa	10.0 ± 1.7Cc	17.8 ± 2.8Cbc	16.5	< 0.01
3 days	37.8 ± 3.6BCb	74.4 ± 4.8Aa	13.3 ± 1.7Cd	23.3 ± 3.3BCc	40.4	< 0.01
4 days	55.6 ± 4.1ABCb	86.7 ± 4.4Aa	28.9 ± 2.0Bc	53.3 ± 3.7ABb	40.6	< 0.01
5 days	70.0 ± 4.7ABa	90.0 ± 3.7Aa	46.7 ± 5.0ABb	67.8 ± 5.2Aa	11.8	< 0.01
6 days	84.4 ± 5.0ABa	92.2 ± 3.6Aa	64.4 ± 7.5ABb	83.3 ± 3.7Aa	5.2	< 0.01
7 days	94.4 ± 3.8Aa	95.6 ± 2.9Aa	75.6 ± 6.0Ab	86.7 ± 2.9Aab	5.0	0.01
*F*	100.0	146.4	125.9	69.5		
*P*	< 0.01	< 0.01	< 0.01	< 0.01		

Within each row, means followed by the same lowercase letter are not significantly different (*df* = 3, 35; Tukey's HSD test at *P* = 0.05). Within each column, means followed by the same uppercase letter are not significantly different (*df* = 9, 89; Tukey's HSD test at *P* = 0.05). Where no letters exist, no significant differences were recorded. Where dashes exist, no statistical analysis was performed

Concerning *O. surinamensis* larvae, main effects were significant between and within exposure intervals (Table 4). The *n*-hexane extract tested at 1000 ppm led to 53.3% mortality after a 2-day exposure, while all the other treatments killed 10.0 – 27.8% (Table 7). The same concentration of the *n*-hexane extract caused the death to 90.0% of *O. surinamensis* larvae the 5^th^ day of the trials. At the end of the bioassays, 94.4 and 95.6% of the individuals died by 500 ppm and 1000 ppm of *n*-hexane extract, respectively. Methanol extract did not exceed 75.6% (500 ppm) and 86.7% (1000 ppm) mortality at the same interval.

#### Adults and larvae of *Trogoderma granarium*

For *T. granarium* adults, between and within exposure intervals main effects and interactions were significant (Table 4). At the 3^rd^ day of the experimentation, mortality ranged between 33.3% (500 ppm *n*-hexane extract) and 64.4% (1000 ppm methanol extract) (Table 8). Both extracts tested at 1000 ppm led to high mortality levels i.e., 94.4% (*n*-hexane extract) and 93.3% (methanol extract) the 6^th^ day; 100.0% (*n*-hexane extract) and 97.8% (methanol extract) the 7^th^ day. The lowest concentration of 500 ppm killed 85.6% (*n*-hexane extract) and 83.3% (methanol extract) at the end of the trials.

**Table 8:** Mean (%) mortality ± standard error (SE) of *T. granarium* adults and larvae after 4 h, 8 h, 16 h, and 1–7 days on wheat treated with *A. oleracea* *n*-hexane and methanol extracts at two different concentrations

Adults	*n*-Hexane extract 500 ppm	*n*-Hexane extract 1000 ppm	Methanol extract 500 ppm	Methanol extract 1000 ppm	*F*	*P*
4 h	0.0 ± 0.0E	0.0 ± 0.0D	0.0 ± 0.0D	0.0 ± 0.0C	-	-
8 h	0.0 ± 0.0Eb	0.0 ± 0.0Db	0.0 ± 0.0Db	3.3 ± 1.7Ca	4.0	0.02
16 h	1.1 ± 1.1Eb	2.2 ± 1.5CDb	0.0 ± 0.0Db	14.4 ± 2.9Ba	16.3	< 0.01
1 day	5.6 ± 1.8Db	6.7 ± 2.9Cb	2.2 ± 1.5Db	21.1 ± 3.9Ba	7.4	< 0.01
2 days	16.7 ± 2.4Cc	24.4 ± 3.8Bbc	32.2 ± 3.2Cab	46.7 ± 4.4Aa	12.2	< 0.01
3 days	33.3 ± 3.7BCb	44.4 ± 5.6ABab	46.7 ± 5.0BCab	64.4 ± 6.0Aa	6.7	< 0.01
4 days	45.6 ± 3.8ABb	77.8 ± 4.6Aa	53.3 ± 5.5ABCb	77.8 ± 6.0Aa	11.8	< 0.01
5 days	54.4 ± 4.1ABc	87.8 ± 3.6Aa	66.7 ± 4.7ABbc	83.3 ± 5.3Aab	11.7	< 0.01
6 days	56.7 ± 4.4ABb	94.4 ± 2.9Aa	80.0 ± 4.7ABa	93.3 ± 3.3Aa	20.1	< 0.01
7 days	85.6 ± 3.8Abc	100.0 ± 0.0Aa	83.3 ± 4.7Ac	97.8 ± 2.2Aab	6.3	< 0.01
*F*	112.1	98.1	253.9	80.4		
*P*	< 0.01	< 0.01	< 0.01	< 0.01		
Larvae						
4 h	0.0 ± 0.0C	0.0 ± 0.0D	0.0 ± 0.0D	0.0 ± 0.0D	-	-
8 h	0.0 ± 0.0C	0.0 ± 0.0D	0.0 ± 0.0D	0.0 ± 0.0D	-	-
16 h	0.0 ± 0.0Cb	4.4 ± 1.8Ca	0.0 ± 0.0Db	0.0 ± 0.0Db	6.4	< 0.01
1 day	3.3 ± 1.7BCb	8.9 ± 2.0Ba	0.0 ± 0.0Db	0.0 ± 0.0Db	11.2	< 0.01
2 days	10.0 ± 3.3ABCb	33.3 ± 3.3Aa	3.3 ± 1.7CDb	7.8 ± 2.8Cb	8.1	< 0.01
3 days	14.4 ± 3.4ABb	37.8 ± 3.6Aa	7.8 ± 2.2BCb	20.0 ± 4.4Bab	6.6	< 0.01
4 days	16.7 ± 4.1ABb	46.7 ± 3.3Aa	16.7 ± 2.9ABb	28.9 ± 4.8ABab	4.8	0.01
5 days	26.7 ± 5.8Aab	50.0 ± 4.1Aa	17.8 ± 2.8ABb	35.6 ± 5.6ABab	3.7	0.02
6 days	27.8 ± 6.0Ab	52.2 ± 3.2Aa	24.4 ± 3.4ABb	50.0 ± 5.5Aa	3.7	0.02
7 days	27.8 ± 6.0Ab	52.2 ± 3.2Aa	27.8 ± 4.7Ab	60.0 ± 5.3Aa	4.1	0.02
*F*	12.6	78.0	21.1	89.5		
*P*	< 0.01	< 0.01	< 0.01	< 0.01		

Within each row, means followed by the same lowercase letter are not significantly different (*df* = 3, 35; Tukey's HSD test at *P* = 0.05). Within each column, means followed by the same uppercase letter are not significantly different (*df* = 9, 89; Tukey's HSD test at *P* = 0.05). Where dashes exist, no statistical analysis was performed

Between and within exposure intervals, concentration, exposure, and exposure × extract were significant in the case of *T. granarium* larvae (Table 4). After a day of exposure, *n*-hexane extract did not exceed 8.9% mortality (1000 ppm), while both methanol concentrations did not cause any deaths (Table 8). Mortalities of *T. granarium* larvae were moderate reaching 52.2% (1000 ppm *n*-hexane extract) and 60.0% (1000 ppm methanol extract) at the end of the bioassays. Both extracts of 500 ppm killed 27.8% of the larvae at the same interval.

#### Adults and larvae of *Tribolium castaneum*

Extract, exposure, and exposure × extract were significant for *T. castaneum* adults between and within exposure intervals (Table 4). No mortality was noticed 16 h after the exposure to both concentrations of *n*-hexane extract and after a day to both doses of methanol extract (Table 9). In general, mortality rates were low for *T. castaneum* adults, not exceeding 15.6 and 18.9% at the end of the trials, for 1000 ppm of *n*-hexane and methanol extracts, respectively.

**Table 9:** Mean (%) mortality ± standard error (SE) of *T. castaneum* adults and larvae after 4 h, 8 h, 16 h, and 1–7 days on wheat treated with *A. oleracea* *n*-hexane and methanol extracts at two different concentrations

Adults	*n*-Hexane extract 500 ppm	*n*-Hexane extract 1000 ppm	Methanol extract 500 ppm	Methanol extract 1000 ppm	*F*	*P*
4 h	0.0 ± 0.0B	0.0 ± 0.0B	0.0 ± 0.0B	0.0 ± 0.0C	-	-
8 h	0.0 ± 0.0B	0.0 ± 0.0B	0.0 ± 0.0B	0.0 ± 0.0C	-	-
16 h	0.0 ± 0.0B	0.0 ± 0.0B	0.0 ± 0.0B	0.0 ± 0.0C	-	-
1 day	1.1 ± 1.1ABab	4.4 ± 1.8ABa	0.0 ± 0.0B	0.0 ± 0.0C	4.1	0.01
2 days	1.1 ± 1.1AB	7.8 ± 3.2AB	4.4 ± 2.4AB	5.6 ± 1.8B	1.7	0.18
3 days	5.6 ± 3.4ABb	8.9 ± 3.5ABab	5.6 ± 2.4ABab	12.2 ± 1.5Aa	3.1	0.04
4 days	6.7 ± 3.3AB	10.0 ± 3.3A	7.8 ± 3.2AB	13.3 ± 1.7A	2.3	0.10
5 days	7.8 ± 3.2AB	12.2 ± 3.2A	8.9 ± 3.1A	14.4 ± 1.8A	1.9	0.15
6 days	12.2 ± 4.9AB	15.6 ± 4.4A	8.9 ± 3.1A	16.7 ± 1.7A	1.7	0.19
7 days	13.3 ± 4.7A	15.6 ± 4.4A	8.9 ± 3.1A	18.9 ± 1.1A	1.8	0.16
*F*	4.1	6.1	5.1	81.4		
*P*	< 0.01	< 0.01	< 0.01	< 0.01		
Larvae						
4 h	0.0 ± 0.0E	0.0 ± 0.0D	0.0 ± 0.0C	0.0 ± 0.0D	-	-
8 h	0.0 ± 0.0E	2.2 ± 1.5D	0.0 ± 0.0C	0.0 ± 0.0D	2.3	0.10
16 h	0.0 ± 0.0Eb	15.6 ± 2.9Ca	0.0 ± 0.0Cb	3.3 ± 1.7Db	20.7	< 0.01
1 day	12.2 ± 2.8Db	31.1 ± 4.6Ba	0.0 ± 0.0Cc	12.2 ± 2.8Cb	20.9	< 0.01
2 days	17.8 ± 2.8CDb	54.4 ± 3.8ABa	2.2 ± 1.5Cc	16.7 ± 3.3BCb	22.0	< 0.01
3 days	33.3 ± 5.0BCa	70.0 ± 5.8Aa	5.6 ± 2.4BCb	30.0 ± 4.1ABa	26.3	< 0.01
4 days	58.9 ± 4.2ABa	82.2 ± 4.3Aa	16.7 ± 4.4ABb	37.8 ± 4.7ABa	13.7	< 0.01
5 days	70.0 ± 5.0ABa	95.6 ± 2.4Aa	21.1 ± 4.8Ab	57.8 ± 4.7Aa	16.1	< 0.01
6 days	83.3 ± 4.7Aa	98.9 ± 1.1Aa	35.6 ± 6.9Ab	68.9 ± 3.5Aa	19.7	< 0.01
7 days	87.8 ± 4.3Aa	100.0 ± 0.0Aa	38.9 ± 8.1Ab	76.7 ± 4.4Aa	16.7	< 0.01
*F*	105.9	105.4	27.9	57.1		
*P*	< 0.01	< 0.01	< 0.01	< 0.01		

Within each row, means followed by the same lowercase letter are not significantly different (*df* = 3, 35; Tukey's HSD test at *P* = 0.05). Within each column, means followed by the same uppercase letter are not significantly different (*df* = 9, 89; Tukey's HSD test at *P* = 0.05). Where no letters exist, no significant differences were recorded. Where dashes exist, no statistical analysis was performed

Regarding *T. castaneum* larvae, main effects and interactions were significant between and within exposure intervals, except for extract × concentration (Table 4). The *n*-hexane extract tested at 1000 ppm provided 54.4% mortality after a 2-day exposure (Table 9). At the 5^th^ day, the same concentration of *n*-hexane extract killed 95.6% of the exposed *T. castaneum* larvae while the other treatments caused 21.1 – 70.0% mortality. At the end of the bioassays, 1000 ppm of the *n*-hexane extract caused the death to all larvae, followed by 500 ppm of *n*-hexane extract (87.8%), 1000 ppm of methanol extract (76.7%) and 500 ppm of methanol extract (38.9%).

#### Adults and larvae of *Tribolium confusum*

Extract, concentration (between exposure intervals) and exposure (within exposure intervals) were significant for *T. confusum* adults (Table 4). No mortality was noted for 16 h, 8 h and 2 days after the exposure to 500 ppm of *n*-hexane extract, 1000 ppm of *n*-hexane extract and both methanol concentrations, respectively (Table 10). At the end of the trials, mortality ranged between 1.1% (500 ppm of methanol extract) and 18.9% (1000 ppm of *n*-hexane extract).

**Table 10:** Mean (%) mortality ± standard error (SE) of *T. confusum* adults and larvae after 4 h, 8 h, 16 h, and 1–7 days on wheat treated with *A. oleracea* *n*-hexane and methanol extracts at two different concentrations

Adults	*n*-Hexane extract 500 ppm	*n*-Hexane extract 1000 ppm	Methanol extract 500 ppm	Methanol extract 1000 ppm	*F*	*P*
4 h	0.0 ± 0.0	0.0 ± 0.0B	0.0 ± 0.0	0.0 ± 0.0B	-	-
8 h	0.0 ± 0.0	0.0 ± 0.0B	0.0 ± 0.0	0.0 ± 0.0B	-	-
16 h	0.0 ± 0.0	2.2 ± 1.5AB	0.0 ± 0.0	0.0 ± 0.0B	2.3	0.10
1 day	1.1 ± 1.1ab	4.4 ± 1.8ABa	0.0 ± 0.0b	0.0 ± 0.0Bb	4.1	0.01
2 days	2.2 ± 1.5ab	7.8 ± 3.6ABa	0.0 ± 0.0b	0.0 ± 0.0Bb	3.7	0.02
3 days	2.2 ± 1.5	8.9 ± 4.6AB	1.1 ± 1.1	2.2 ± 1.5AB	1.3	0.29
4 days	4.4 ± 2.4	11.1 ± 4.6AB	1.1 ± 1.1	4.4 ± 1.8AB	1.7	0.18
5 days	4.4 ± 2.4	14.4 ± 6.7AB	1.1 ± 1.1	5.6 ± 2.4AB	1.8	0.17
6 days	5.6 ± 3.4ab	18.9 ± 6.1Aa	1.1 ± 1.1b	1.1 ± 1.1b	4.3	0.01
7 days	6.7 ± 3.3ab	18.9 ± 6.1Aa	1.1 ± 1.1b	7.8 ± 2.8Aab	4.0	0.02
*F*	1.7	3.9	0.6	4.5		
*P*	0.12	< 0.01	0.83	< 0.01		
Larvae						
4 h	0.0 ± 0.0E	0.0 ± 0.0D	0.0 ± 0.0D	0.0 ± 0.0D	-	-
8 h	0.0 ± 0.0E	0.0 ± 0.0D	0.0 ± 0.0D	2.2 ± 1.5D	2.3	0.10
16 h	0.0 ± 0.0Eb	3.3 ± 1.7Dab	10.0 ± 6.7CDab	11.1 ± 3.5Ca	3.4	0.03
1 day	7.8 ± 1.5D	11.1 ± 3.1C	16.7 ± 7.1BC	17.8 ± 3.2BC	0.7	0.55
2 days	27.8 ± 3.2C	33.3 ± 5.0B	23.3 ± 6.2AB	31.1 ± 3.5AB	1.6	0.21
3 days	46.7 ± 3.7BCab	58.9 ± 6.1ABa	32.2 ± 6.4ABc	33.3 ± 3.3ABbc	7.1	< 0.01
4 days	64.4 ± 4.1ABa	70.0 ± 6.5ABa	36.7 ± 7.6Ab	40.0 ± 3.7ABb	9.5	< 0.01
5 days	76.7 ± 5.3ABa	77.8 ± 4.9Aa	40.0 ± 7.6Ab	45.6 ± 4.7Ab	12.1	< 0.01
6 days	83.3 ± 4.4ABa	84.4 ± 2.9Aa	48.9 ± 7.5Ab	66.7 ± 4.7Aa	11.7	< 0.01
7 days	85.6 ± 3.8Aa	88.9 ± 2.6Aa	57.8 ± 8.3Ab	80.0 ± 4.1Aa	8.7	< 0.01
*F*	239.2	93.1	36.0	44.4		
*P*	< 0.01	< 0.01	< 0.01	< 0.01		

Within each row, means followed by the same lowercase letter are not significantly different (*df* = 3, 35; Tukey's HSD test at *P* = 0.05). Within each column, means followed by the same uppercase letter are not significantly different (*df* = 9, 89; Tukey's HSD test at *P* = 0.05). Where no letters exist, no significant differences were recorded. Where dashes exist, no statistical analysis was performed

For *T. confusum* larvae, concentration, exposure, exposure × extract and exposure × extract × concentration were significant between and within exposure intervals (Table 4). The 3^rd^ day of the experimentations, 1000 ppm of *n*-hexane extract killed 58.9% of the larvae while 1000 ppm of methanol extract did not exceed 33.3% mortality (Table 10). At the termination of the trials, 1000 ppm of *n*-hexane extract caused 88.9% mortality while 500 ppm 85.6%. At the same interval, 57.8 and 80.0% of larvae were dead after their exposure to 500 ppm and 1000 ppm of methanol extract, respectively.

#### Adults and larvae of *Tenebrio molitor*

As far as *T. molitor* adults are concerned, the type of extract, concentration, exposure, and exposure × extract were significant between and within exposure intervals (Table 4). For 8 and 16 h, no mortality was recorded for *n*-hexane and methanol concentrations respectively (Table 11). Testing 500 ppm and 1000 ppm of the *n*-hexane extract, mortality rates remained low for the whole experimental interval, not exceeding 13.3 and 24.4%, respectively. In the case of the methanol extract, all adults died after 5 days (1000 ppm) while after a 7-day exposure 91.1% of the individuals were dead (500 ppm).

**Table 11:** Mean (%) mortality ± standard error (SE) of *T. molitor* adults and larvae after 4 h, 8 h, 16 h, and 1–7 days on wheat treated with *A. oleracea* *n*-hexane and methanol extracts at two different concentrations

Adults	*n*-Hexane extract 500 ppm	*n*-Hexane extract 1000 ppm	Methanol extract 500 ppm	Methanol extract 1000 ppm	*F*	*P*
4 h	0.0 ± 0.0B	0.0 ± 0.0D	0.0 ± 0.0D	0.0 ± 0.0E	-	-
8 h	0.0 ± 0.0B	0.0 ± 0.0D	0.0 ± 0.0D	0.0 ± 0.0E	-	-
16 h	0.0 ± 0.0Bb	0.0 ± 0.0Db	3.3 ± 1.7Dab	4.4 ± 1.8Da	3.6	0.02
1 day	1.1 ± 1.1Bc	3.3 ± 1.6CDbc	8.9 ± 2.0Cab	10.0 ± 1.7Ca	7.5	< 0.01
2 days	2.2 ± 1.5ABb	5.6 ± 1.8BCDb	36.7 ± 6.0Ba	43.3 ± 4.7Ba	28.9	< 0.01
3 days	3.3 ± 1.7ABb	6.7 ± 2.4ABCDb	63.3 ± 6.5ABa	68.9 ± 4.2ABa	34.0	< 0.01
4 days	4.4 ± 1.8ABb	8.9 ± 2.0ABCb	72.2 ± 6.2ABa	93.3 ± 2.9Aa	36.2	< 0.01
5 days	5.6 ± 2.4ABb	12.2 ± 2.8ABCb	80.0 ± 5.8ABa	100.0 ± 0.0Aa	29.8	< 0.01
6 days	8.9 ± 3.5ABb	17.8 ± 4.0ABb	86.7 ± 4.4Aa	100.0 ± 0.0Aa	20.0	< 0.01
7 days	13.3 ± 4.4Ab	24.4 ± 3.8Ab	91.1 ± 2.6Aa	100.0 ± 0.0Aa	16.5	< 0.01
*F*	3.8	9.6	101.2	138.5		
*P*	< 0.01	< 0.01	< 0.01	< 0.01		
Larvae						
4 h	0.0 ± 0.0D	0.0 ± 0.0D	0.0 ± 0.0D	0.0 ± 0.0D	-	-
8 h	0.0 ± 0.0D	0.0 ± 0.0D	0.0 ± 0.0D	0.0 ± 0.0D	-	-
16 h	0.0 ± 0.0D	0.0 ± 0.0D	0.0 ± 0.0D	0.0 ± 0.0D	-	-
1 day	0.0 ± 0.0D	1.1 ± 1.1D	0.0 ± 0.0D	2.2 ± 1.5C	1.3	0.28
2 days	2.2 ± 1.5CDc	16.7 ± 3.7Cb	0.0 ± 0.0Dc	55.6 ± 2.4Ba	54.6	< 0.01
3 days	4.4 ± 1.8Cc	44.4 ± 4.4Bab	18.9 ± 2.0Cb	76.7 ± 3.7ABa	40.6	< 0.01
4 days	17.8 ± 3.2Bb	76.7 ± 5.0ABa	44.4 ± 3.8Ba	88.9 ± 3.9ABa	20.0	< 0.01
5 days	44.4 ± 3.4Ac	95.6 ± 2.4Aa	66.7 ± 2.9Ab	93.3 ± 2.9ABa	54.6	< 0.01
6 days	68.9 ± 4.2Ab	97.8 ± 2.2Aa	75.6 ± 3.8Ab	94.4 ± 2.4ABa	16.1	< 0.01
7 days	86.7 ± 3.3Abc	100.0 ± 0.0Aa	80.0 ± 4.4Ac	97.8 ± 1.5Aab	10.2	< 0.01
*F*	82.9	216.7	1659.0	375.9		
*P*	< 0.01	< 0.01	< 0.01	< 0.01		

Within each row, means followed by the same lowercase letter are not significantly different (*df* = 3, 35; Tukey's HSD test at *P* = 0.05). Within each column, means followed by the same uppercase letter are not significantly different (*df* = 9, 89; Tukey's HSD test at *P* = 0.05). Where no letters exist, no significant differences were recorded. Where dashes exist, no statistical analysis was performed

Regarding *T. molitor* larvae, all main effects and interactions were significant between and within exposure intervals, except extract × concentration (Table 4). During the first 2 days of the trials, 1000 ppm of methanol extract killed 55.6% of *T. molitor* larvae while mortalities of the other treated treatments ranged between 0.0 and 16.7% (Table 11). The 5^th^ day of the experiments, both 1000 ppm of *n*-hexane and methanol extracts provided high mortalities (95.6 and 93.3% respectively). At the termination of the bioassays, 500 and 1000 ppm of *n*-hexane extract and 500 and 1000 ppm of methanol extract caused the death to 86.7, 100.0, 80.0, and 97.8% of the exposed larvae, respectively.

#### Adults and larvae of *Alphitobius diaperinus*

Regarding *A. diaperinus* adults, between and within exposure intervals none of the main effects or interactions were significant (Table 4). No mortality was recorded in the case of 500 ppm of the *n*-hexane extract the whole experimental period, while for 6 days mortality was 0.0% for 1000 ppm of *n*-hexane extract and 500 ppm of methanol extract (Table 12). The higher concentration of methanol extract killed 3.3% of adults 7 days post-exposure.

**Table 12:** Mean (%) mortality ± standard error (SE) of *A. diaperinus* adults and larvae after 4 h, 8 h, 16 h, and 1–7 days on wheat treated with *A. oleracea* *n*-hexane and methanol extracts at two different concentrations

Adults	*n*-Hexane extract 500 ppm	*n*-Hexane extract 1000 ppm	Methanol extract 500 ppm	Methanol extract 1000 ppm	*F*	*P*
4 h	0.0 ± 0.0	0.0 ± 0.0	0.0 ± 0.0	0.0 ± 0.0	-	-
8 h	0.0 ± 0.0	0.0 ± 0.0	0.0 ± 0.0	0.0 ± 0.0	-	-
16 h	0.0 ± 0.0	0.0 ± 0.0	0.0 ± 0.0	0.0 ± 0.0	-	-
1 day	0.0 ± 0.0	0.0 ± 0.0	0.0 ± 0.0	1.1 ± 1.1	1.0	0.41
2 days	0.0 ± 0.0	0.0 ± 0.0	0.0 ± 0.0	1.1 ± 1.1	1.0	0.41
3 days	0.0 ± 0.0	0.0 ± 0.0	0.0 ± 0.0	1.1 ± 1.1	1.0	0.41
4 days	0.0 ± 0.0	0.0 ± 0.0	0.0 ± 0.0	1.1 ± 1.1	1.0	0.41
5 days	0.0 ± 0.0	0.0 ± 0.0	0.0 ± 0.0	1.1 ± 1.1	1.0	0.41
6 days	0.0 ± 0.0	0.0 ± 0.0	0.0 ± 0.0	2.2 ± 1.5	2.3	0.10
7 days	0.0 ± 0.0	1.1 ± 1.1	1.1 ± 1.1	3.3 ± 1.7	1.5	0.24
*F*	-	1.0	1.0	1.0		
*P*	-	0.45	0.45	0.46		
Larvae						
4 h	0.0 ± 0.0C	0.0 ± 0.0E	0.0 ± 0.0D	0.0 ± 0.0D	-	-
8 h	0.0 ± 0.0C	0.0 ± 0.0E	0.0 ± 0.0D	0.0 ± 0.0D	-	-
16 h	0.0 ± 0.0Cb	5.6 ± 1.8Da	0.0 ± 0.0Db	2.2 ± 1.5CDab	5.3	0.01
1 day	1.1 ± 1.1Cb	16.7 ± 2.9Ca	6.7 ± 1.7Cab	6.7 ± 3.3Cab	6.5	< 0.01
2 days	21.1 ± 3.9Bb	44.4 ± 4.8Ba	24.4 ± 2.4Bab	36.7 ± 4.4Bab	4.1	0.01
3 days	56.7 ± 4.7Aab	71.1 ± 5.4ABa	46.7 ± 2.9Ab	65.6 ± 4.4ABa	5.3	0.01
4 days	73.3 ± 5.0Ab	92.2 ± 2.8ABa	60.0 ± 3.3Ab	91.1 ± 4.8Aa	12.5	< 0.01
5 days	85.6 ± 4.1Aa	100.0 ± 0.0Aa	64.4 ± 4.1Ab	98.9 ± 1.1Aa	23.8	< 0.01
6 days	94.4 ± 2.4Aa	100.0 ± 0.0Aa	75.6 ± 3.4Ab	100.0 ± 0.0Aa	26.0	< 0.01
7 days	100.0 ± 0.0Aa	100.0 ± 0.0Aa	83.3 ± 5.0Ab	100.0 ± 0.0Aa	10.2	< 0.01
*F*	206.2	116.3	197.5	114.4		
*P*	< 0.01	< 0.01	< 0.01	< 0.01		

Within each row, means followed by the same lowercase letter are not significantly different (*df* = 3, 35; Tukey's HSD test at *P* = 0.05). Within each column, means followed by the same uppercase letter are not significantly different (*df* = 9, 89; Tukey's HSD test at *P* = 0.05). Where no letters exist, no significant differences were recorded. Where dashes exist, no statistical analysis was performed

Concerning *A. diaperinus* larvae, concentration was significant between exposure intervals while within exposure intervals all main effects and interaction were significant (Table 4). Mortality was moderate the 3^rd^ day of the trials, ranging from 46.7% (500 ppm of methanol extract) to 71.1% (1000 ppm of *n*-hexane extract) (Table 12). Complete mortality was noted for 1000 ppm of *n*-hexane extract, 1000 ppm of methanol extract and 500 ppm of *n*-hexane extract after 5, 6, and 7 days of exposure, respectively. The methanol extract tested at 500 ppm did not exceed 83.3% mortality at the end of the trials.

## Discussion

Regarding the screening of *A. oleracea* extraction, spilanthol has been reported to be extracted by various solvents. *n*-Hexane (Ramsewak et al. 1999), ethanol (Simas et al. 2013), and methanol are the most frequently described (Mbeunkui et al. 2011). Several studies also report the use of combination of solvents to extract the compound, such as ethanol:*n*-hexane (3:7, v/v) (Costa et al. 2013), or ethanol:water (7:3, v/v) (Martins et al. 2012).

On the other hand, regarding the screening of extraction techniques, earlier research reported the use of microwave-assisted extraction (Franca et al. 2016), supercritical CO_2_ extraction (Dias et al. 2012), and Soxhlet (Bakondi et al. 2019). For instance, Franca et al. (2016) compared microwave-assisted extraction with normal maceration, demonstrating that the use of microwave allowed obtaining the highest amount of spilanthol. Bellumori et al. (2022) recently screened three different *A. oleracea* extraction procedures using ethanol 80% v/v as extractive solvent:sonication at 60 °C for 10 min, magnetic stirring for 50 min followed by sonication for 10 min, and sonication for 10 min at room temperature. Their study demonstrated that sonication at 60 °C for 10 min was the best extractive technique and that if a fractionation step with *n*-hexane was applied to the obtained ethanolic extract, an enriched *N*-alkylamides fraction could be obtained. Bellumori et al. (2022) suggested that *n*-hexane gives an extract enriched in spilanthol and this is linear with the results presented in our study, even if a different extractive approach was used. According to Bellumori et al. (2022), magnetic stirring is not the best spilanthol extractive technique, as also evident from our study. In fact, magnetic stirring resulted one of the worst spilanthol extractive techniques (Table 2). These results were also linear with those of Grymel et al. (2023). Indeed, between Soxhlet extraction, magnetic stirring at high temperature and room temperature, and maceration at room temperature, Soxhlet resulted the best extraction technique for the highest recovery of the *N*-alkylamide from the biomass. Regarding the identification of the 6 *N*-alkylamides, our results are quite comparable to that of Bae et al. (2010), even if they identifided 3 more *N*-alkylamides from a 75% ethanol *A. oleracea* extract, namely (*2E*,*4Z*)-*N*-isobutly-2,4-undecadiene-8,10-diynamide, (*2E*,*7Z*)-*N*-isobutyl-2,7-tridecadiene-10,12-diynamide, (*2E*,*4E*,*8Z*,*10E*)-*N*-isobutyl-dodeca-2,4,8,10-tetraenamide (Bae et al. 2010). Furthermore, Cheng et al. (2015), isolated a new *N*-alkylamide, identified as (*2E*,*5Z*)-*N*-isobutylundeca-2,5-diene-8,10-diynamide in an ethanol extract that was not found in our study. Moreover, *N*-phenethyl-2,3-epoxy-6,8-nonadiynamide, (*2E*,*4Z*)-*N*-isobutyl-2,4-undecadiene-8,10-diynamide, (*2E*)-*N*-(2-methylbutyl)-2-undecene-8,10-diynamide, (*2E*,*7Z*)-*N*-isobutyl-2,7-tridecadiene-10,12-diynamide, and (*2E*,*4E*,*8Z*,*10Z*)-*N*-isobutyl-dodeca-2,4,8,10-tetraenamide were identified by Boonen et al. (2010) in an ethanol extract, differently from our results.

Regarding the *A. oleracea* toxicity on arthropod pests, our results indicate the high effectiveness of the *n*-hexane and methanol extracts derived from *A. oleracea* against most of the tested pests and developmental stages. The *n*-hexane extract was more effective than the methanol extract, in almost all tested cases. This trend has been outlined earlier by Araújo et al. (2020), who tested three extracts from *A. oleracea* (i.e., *n*-hexanic, hydroethanolic, and methanolic) against *Aedes aegypti* Linnaeus (Diptera: Culicidae) larvae. The 10, 12, 20, and 30 μg/mL *n*-hexanic extract was more efficient, than the same concentrations of the hydroethanolic and methanolic extracts, after 48 h of exposure. Furthermore, the *n*-hexane extract killed more *T. absoluta* than the ethanol extract (Moreno et al. 2012). Marchesini et al. (2020) suggested that the different efficacies of *A. oleracea* extracts can be attributed to their content in spilanthol. In fact, the highest activity of the *n*-hexane extract could be ascribed to its highest concentration of spilanthol (24.3 ± 1.3 g/100 g DE), if compared with the methanolic one (7.6 ± 0.3 g/100 g DE) (Table 3). These authors found that the more spilanthol an extract contained (0.0 – 100.0% spilanthol), the higher the efficacy against *R. microplus* larvae (0.0 – 100.0% mortality) is observed. The hexane extract used by Castro et al. (2014) against *R. microplus* larvae, having spilanthol as main ingredient, provided extremely low lethal doses, i.e., 0.8 mg/mL for LC_95_. In addition, Pandey et al. (2011) studied the larvicidal effects of compounds derived from *A. oleracea* against *Anopheles stephensi* Liston (Diptera: Culicidae) larvae. Apart from spilanthol, the authors found two additional larvicidal compounds: (2*E*)-*N*-(2-methylbutyl)-2-undecene-8,10-diynamide and undeca-2*E*,7*Z*,9*E*-trienoic acid isobutylamide. Other products derived from *A. oleracea,* such as the whole essential oil (EO) and its nanoemulsion (NE), have been utilized for managing a relatively broad number of insect species of public health and agricultural importance. For example, Benelli et al. (2019) documented high acute toxicity of *A. oleracea* EO, containing little amount of spilanthol, against *Musca domestica* Linnaeus (Diptera: Muscidae) adults, *Culex quinquefasciatus* Say (Diptera: Culicidae) larvae, and *Spodoptera littoralis* (Boisduval) (Lepidoptera: Noctuidea) larvae. On the other hand, spilanthol alone exhibited the highest efficacy against *C. quinquefasciatus* larvae, followed closely by the *n*-hexane extract of *A. oleracea* and the *A. oleracea* EO, while the *A. oleracea* EO-based NE was not as effective as the previous products (Spinozzi et al. 2021).

Herein we offer novel results on the potential application of *A. oleracea* extracts for managing foodstuff arthropod pests. During the present study we observed different susceptibility among the tested pests and their developmental stages. For instance, 500 ppm of the *n*-hexane extract killed 100.0% of *A. diaperinus* larvae but they did not kill any *A. diaperinus* adults, at the end of the trials. The insecticidal activity displayed by the extracts is mainly linked to the presence of spilanthol. The mechanism of action of this *N*-alkylamide seems to be linked to the affection of the central nervous system, but it was also noticed that it can disrupt the processes of histolysis of larval tissues (Moreno et al. 2012). For instance, Saraf and Dixit (2002) reported a high pupal mortality of several mosquito species after spilanthol treatment, supporting the hypothesis that the *N*-alkylamide acts on histolysis and histogenesis processes. Consequently, it could be hypothesized that the action of *A. oleracea* extracts reported in our work is ascribed both to feeding toxicity and contact toxicity. Further research is needed to confirm this hypothesis.

Concerning setae, previous studies have documented that they can serve the insect as a protective barrier between its body and the treated surface, while the absence of setae can make an insect susceptible (Peterson 1948, 1951; Hadaway 1956; Carlson and Ball 1962; Athanassiou et al. 2006). This statement can be supported by the results of this study since *T. granarium* larvae (that have many large setae) were more tolerant than *T. confusum, T. castaneum, A. diaperinus, O. surinamensis,* and *T. molitor* larvae (that have few small setae) (Peterson 1951; Rees 2004).

Due to the insect characteristics, pesticide susceptibility/tolerance trends appear for each insect species/stage. For example, previous studies reported the tolerance and susceptibility of *T. confusum* and *T. castaneum* adults and larvae respectively, after their exposure to the EO-based NEs from *Hazomalania voyronii* (Jum.) Capuron, *Smyrnium olusatrum* L. (isofuranodiene extracted from EO), and *Mentha longifolia* (L.) Huds. applied on wheat (Kavallieratos et al. 2021a, b, 2022c). These findings are well aligned with the current study, since both *A. oleracea* extracts provided low mortalities to *Tribolium* spp. adults but high mortalities to *Tribolium* spp. larvae. One of the most important findings of the present study was the high mortality levels caused by both *A. oleracea* extracts against *T. molitor* larvae, which reached 100.0% and 97.8% after a 7-day exposure to the *n*-hexane and methanol extracts, respectively. *Tenebrio molitor* larvae are reported as difficult to be managed (Kavallieratos et al. 2021a, b, 2022c). In some rare cases, *T. molitor* larvae exhibit susceptibility to certain compounds or entomopathogenic fungi isolates (Ntalli et al. 2021; Eski and Murat Gezgin 2022). For example, (*E*)-2-decenal and 2-undecanone killed 80.0 and 87.8% of *T. molitor* larvae, respectively, while *trans*-anethole only 16.7% at the end of the trial (Ntalli et al. 2021). Similarly, the isolate BL8 of *Beauveria bassiana* (Bals. -Criv.) Vuill. and the isolates BL23 and BL24 of *Metarhizium anisopliae* (Metchnikoff) Sorokin provided 100.0% mortality of *T. molitor* larvae, while other isolates, i.e., BL1 of *B. bassiana* did not exceed 17.5% mortality (Eski and Murat Gezgin 2022). Interestingly, only the methanol *A. oleracea* extract caused elevated mortality to *T. molitor* adults. We assume that this stage is tolerant to *A. oleracea* *n*-hexane extract constituents since adults represent a very susceptible stage to numerous synthetic and natural insecticides (Kavallieratos et al. 2019b, 2021a, b, 2022c). Regarding *A. diaperinus,* larvae are susceptible to many insecticides, i.e., d-tetramethrin+piperonyl butoxide+acetamiprid, chlorfenapyr, deltamethrin, and etofenprox (applied on concrete), as well as pirimiphos-methyl and *Carlina acaulis* L. EO (applied on wheat) (Kavallieratos et al. 2022a, b, d, e). The adults, on the other hand, exhibit various susceptibility/tolerance levels. For instance, *A. diaperinus* adults did not exceed 23.3, 25.6, and 31.1% mortality when they were exposed to pirimiphos-methyl, *C. acaulis* EO, and deltamethrin respectively (Kavallieratos et al. 2022d, e). In contrast, chlorfenapyr killed 97.8% of *A. diaperinus* adults (Kavallieratos et al. 2022b). The tested extracts of the current study barely caused mortality to this stage. Thus, there is no holistic trend among these four tenebrionids, while in some cases the insecticidal susceptibility/tolerance varies even among species and developmental stage.

Concerning *C. ferrugineus*, Ikawati et al. (2020) observed that adults were more susceptible than larvae after their exposure to *Citrus hystrix* DC., *Euodia suaveolens* (Hochr.) Bakh. f., *Cinnamomum verum* J.Presl, *Syzygium aromaticum* (L.) Merr. & L.M.Perry, and *Cymbopogon nardus* (L.) Rendle EOs, in fumigant bioassays. Here, both *C. ferrugineus* stages exhibited high levels of susceptibility, but adults reached quicker 100.0% mortality at all tested extracts and concentrations than larvae which needed more time of exposure to die. *Oryzeaphilus surinamensis* and *T. granarium* stages follow the general trends that have already been observed in this study. Both stages of *O. surinamensis* are susceptible to several green insecticides like the *C. acaulis* and *M. longifolia* EOs (Kavallieratos et al. 2022c, e), while larvae of *T. granarium* are more tolerant than the adults (Kavallieratos et al. 2017, 2022f; Kousar et al. 2021; Ali et al. 2022; Saad et al. 2022). *Acarus siro* life stages do not follow a trend since nymphs exhibited higher tolerance than adults when exposed to *A. oleracea* extracts. However, previous studies reported high susceptibility of both stages when exposed to *C. acaulis* EO (Kavallieratos et al. 2022c), or similar susceptibility when exposed to three Apiaceae EO-based NEs (Kavallieratos et al. 2022f). Therefore, the efficacies of the tested *n*-hexane and methanol extracts of *A. oleracea* are characterized by great complexity depending on species/stage.

Apart from the exceptional pesticidal effects of *A. oleracea* extracts against most of the tested arthropod pest species and stages, it is worth mentioning that they are non-toxic towards non target organisms, such as *Chlorella vulgaris* Beijerinck (Chlorellales: Chlorellaceae) (Araújo et al. 2020), or have low risk towards *Tetragonisca angustula* (Latr.) (Hymenoptera: Apidae) and *Solenopsis saevissima* (Smith) (Hymenoptera: Formicidae) (Moreno et al. 2012, but see Giunti et al. 2022 for non-target effects of botanicals). Moreover, this plant has achieved great interest as nutraceutical product and herbal medicine, gaining many patent applications in this field (Sut et al. 2020). In addition, *A. oleracea* aerial parts are listed in the BELFRIT list, which defines a series of plants appropriate for their utilization in food supplements (Cousyn et al. 2013). The plant species is also included in the list of botanicals for use in food supplements in Italy (Italian Ministry of Health 2018). This evidence allows the classification of *A. oleracea* extracts as potentially safe and eco-friendly green pesticides.

Overall, the main findings of this study represent the scientific basis to open new perspectives of use of *A. oleracea* in the agrochemical industry. *Acmella oleracea* is a crop that has recently been much cultivated all around the world due to its applications in the pharmaceutical, nutraceutical, and cosmetic markets. Thus, the current supply chain may warrantee enough material to be also processed by the agrochemical industry. Our study showed that the *n*-hexane and methanol extracts from aerial parts, which are rich in spilanthol and other *N*-alkylamides, were effective against *C. ferrugineus* adults, *A. diaperinus* larvae, *C. ferrugineus* larvae, *T. granarium* adults, *T. molitor* larvae, *O. surinamensis* adults, *O. surinamensis* larvae, *A. siro* adults, *T. confusum* larvae, and *T. castaneum* larvae. Both concentrations of the methanol extract provided high mortalities to *T. molitor* adults, while both extracts provided moderate death to *A. siro* nymphs and *T. granarium* larvae. In contrast, these green pesticides are not efficient against *A. diaperinus*, *T. confusum*, and *T. castaneum* adults. Further research on these extracts, their fractions and spilanthol, as well as on their modes of action (Jankowska et al. 2018) and the effectiveness of encapsulated formulations (e.g., micro- and nanoemuslsions, Pavoni et al. 2019) are still required to assess their pesticidal potential when applied on additional durable food commodities.

## Supplementary Information

Below is the link to the electronic supplementary material.

Supplementary file1 (DOCX 166 KB)

## Data Availability

The datasets used and/or analysed during the current study are available from the corresponding author on reasonable request.
