# Supplementary material for: Acmella oleracea extracts as green pesticides against eight arthropods attacking stored products
Source: Environ Sci Pollut Res Int. 2023 Aug 5;30(41):94904–27. doi: 10.1007/s11356-023-28577-8 (PMC10468743; doi:10.1007/s11356-023-28577-8)
Supplement: Supplementary file 1 — Supplementary file1 (DOCX 166 KB) [file 11356_2023_28577_MOESM1_ESM.docx]

**Supplementary material**

| **Table S1** Yield obtained for the *A. oleracea* screening of solvents and of extraction techniques | |
| --- | --- |
| Solvent^1^ | Yield (%, w/w)^2^ ± SD^3^ |
| *n*-hexane | 2.7 ± 0.3 |
| ethanol | 3.9 ± 0.1 |
| methanol | 11.0 ± 0.1 |
| dichloromethane | 5.5 ± 0.1 |
| petroleum ether | 1.9 ± 0.2 |
| Ethyl acetate | 4.2 ± 0.2 |
| Extraction technique |  |
| Ultrasound bath (1 h) | 10.0 ± 0.3 |
| Ultrasound bath (3 h) | 11.0 ± 1.4 |
| Ultrasound extractor | 7.6 ± 0.4 |
| Magnetic stirring (1 h) | 12.0 ± 0.3 |
| Magnetic stirring (3 h) | 12.0 ± 0.6 |
| Soxhlet | 26.0 ± 0.7 |
| ^1^ Solvent used for the extraction of the plant material in an ultrasound bath for 1 h at room temperature (plant/solvent ratio of 1:10 (w/v)). ^2^ Relative yield values (%) are mean of two independent analyses. ^3^ SD, standard deviation | |

| **Table S2** Method repeatability evaluation | | | | | | |
| --- | --- | --- | --- | --- | --- | --- |
| Concentration (mg/L) | Area | Mean area^1^ | SD intraday^2^ | SD interday^3^ | RSD% intraday^4^ | RSD % interday^5^ |
| 1700 | 11153.7 | 11121.9 | 45.0 |  | 0.40 |  |
|  | 11090.1 |  |  |  |  |  |
| 850 | 5612.9 | 5622.5 | 2.2 | 9.833 | 0.04 | 0.32 |
|  | 5616.0 |  |  |  |  | 0.07 |
|  | 5638.3 |  |  |  |  |  |
|  | 5621.5 |  |  |  |  |  |
|  | 5623.8 |  |  |  |  |  |
| 170 | 1140.7 | 1140.6 | 0.1 |  | 0.01 |  |
|  | 1140.5 |  |  |  |  |  |
| 85 | 596.9 | 596.9 | 0.0 |  | 0 |  |
|  | 596.9 |  |  |  |  |  |
| 17 | 119.8 | 119.9 | 0.1 |  | 0.12 |  |
|  | 120.0 |  |  |  |  |  |
| 8.5 | 54.6 | 55.0 | 0.6 |  | 5.15 |  |
|  | 55.4 |  |  |  |  |  |
| 1.7 | 10.9 | 11.1 | 0.3 |  | 2.86 |  |
|  | 11.3 |  |  |  |  |  |
| ^1^ Mean area is the mean of two independent analyses. ^2^ SD intraday, SD calculated injecting each standard solution 3 times in HPLC in the same day (intraday). ^3^ SD interday, standard deviation calculated injecting 850 mg/L solution 3 times in 3 consecutive days (inter-day). ^4^ RSD% intraday, relative SD calculated for each standard solution injected in the same day (intraday). ^5^ RSD% intraday, relative SD calculated injecting 850 mg/L solution 3 times in 3 consecutive days (inter-day) | | | | | | |


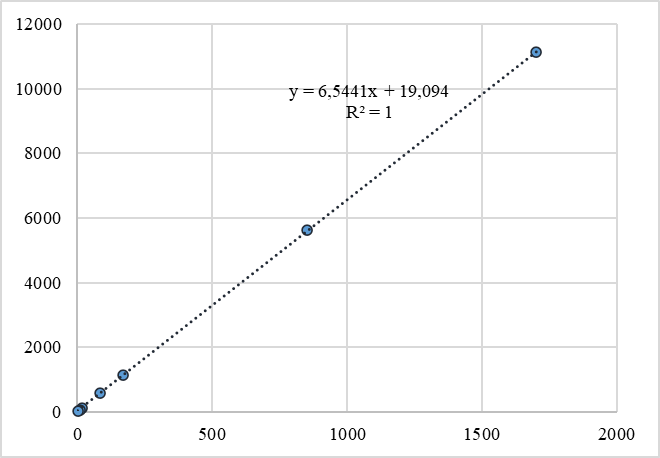


**Fig. S1** Calibration curve of spilanthol obtained through HPLC-DAD-MS analysis


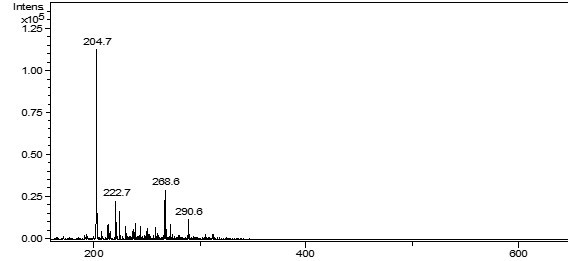


**Fig. S2** (2Z)-N-isobutyl-2-nonene-6,8-diynamide (A1) MS spectrum


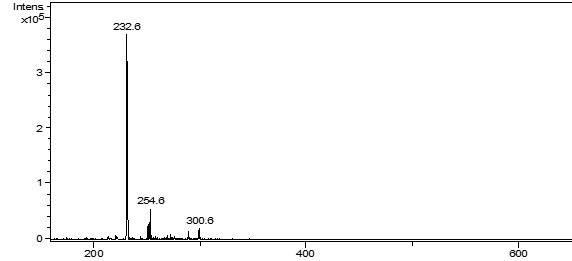


**Fig. S3** (*2E*)-*N*-isobutyl-2-undecene-8,10-diynamide (A2) MS spectrum


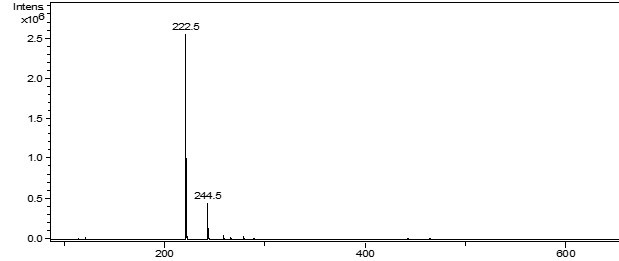


**Fig. S4** (2E,6Z,8E)-N-isobutyl-2,6,8-decatrienamide or spilanthol (A3) MS spectrum


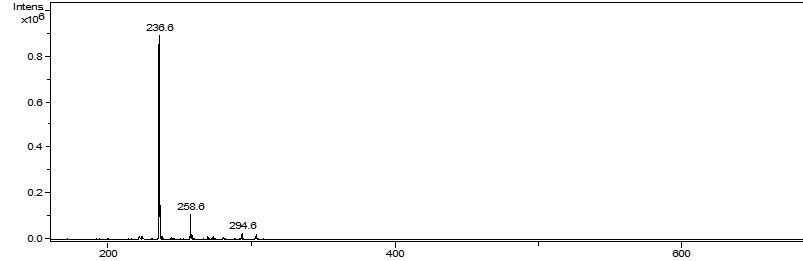


**Fig. S5** (*2E*,*6Z*,*8E*)-*N*-(2-methylbutyl)-2,6,8-decatrienamide (A6) MS spectrum
